# Being good to look good: Self‐reported moral character predicts moral double standards among reputation‐seeking individuals

**DOI:** 10.1111/bjop.12608

**Published:** 2022-11-04

**Authors:** Mengchen Dong, Tom R. Kupfer, Shuai Yuan, Jan‐Willem van Prooijen

**Affiliations:** ^1^ Vrije Universiteit Amsterdam Amsterdam The Netherlands; ^2^ Max Planck Institute for Human Development Berlin Germany; ^3^ Nottingham Trent University Nottingham UK; ^4^ University of Amsterdam Amsterdam The Netherlands

**Keywords:** hypocrisy, moral character, morality, reputation, status

## Abstract

Moral character is widely expected to lead to moral judgements and practices. However, such expectations are often breached, especially when moral character is measured by self‐report. We propose that because self‐reported moral character partly reflects a desire to appear good, people who self‐report a strong moral character will show moral harshness towards others and downplay their own transgressions—that is, they will show greater moral hypocrisy. This self‐other discrepancy in moral judgements should be pronounced among individuals who are particularly motivated by reputation. Employing diverse methods including large‐scale multination panel data (*N* = 34,323), and vignette and behavioural experiments (*N* = 700), four studies supported our proposition, showing that various indicators of moral character (Benevolence and Universalism values, justice sensitivity, and moral identity) predicted harsher judgements of others' more than own transgressions. Moreover, these double standards emerged particularly among individuals possessing strong reputation management motives. The findings highlight how reputational concerns moderate the link between moral character and moral judgement.

**Practitioner points**

- Self‐reported moral character does not predict actual moral performance well.
- Good moral character based on self‐report can sometimes predict strong moral hypocrisy.
- Good moral character based on self‐report indicates high moral standards, while only for others but not necessarily for the self.
- Hypocrites can be good at detecting reputational cues and presenting themselves as morally decent persons.

## BACKGROUND

From Aristotle's virtue ethics onwards, philosophers, social scientists, and educators have long deemed that moral character paves the way to moral judgements and practices (Aquino & Reed, 2002; Kamtekar, 2004; Walker et al., 1987; Walker & Frimer, 2007). The development of moral character, including, for example, endorsement of moral values, sensitivity to (in)justice‐related incidents, and identification with morality as an integral part of the self‐concept, is seen as a fundamental foundation of civic education (Althof & Berkowitz, 2006; Walker et al., 1987). Researchers and practitioners largely employ self‐reported measures, to estimate whether a person possesses a good versus bad moral character, and to infer the person's likelihood of making ethical versus unethical decisions (Aquino & Reed, 2002; DeCelles et al., 2012; Lee et al., 2008). However, self‐reported moral character has not always strongly predicted laudable moral judgements or behaviours. For example, a meta‐analysis based on 111 studies suggested only a small effect size of self‐reported moral identity on actual moral behaviours (Hertz & Krettenauer, 2016). People who feel themselves as morally superior to others do not necessarily enact more moral behaviours (Tappin & McKay, 2019). Their moral identity predicts more public claims but not private acts of integrity (Dong et al., 2019).

We propose that self‐reported moral character is not always strongly linked to morally laudable judgements and behaviours because people report having a strong moral character partly to *appear* good. Taking this further, in the current research we test the hypothesis that people who report having a strong moral character—especially those motivated by reputation—are more likely to display *moral double standards*, in that they evaluate others' transgressions more harshly than their own transgressions. Below we present our reasoning in greater detail.

### Self‐reported moral character and reputation management

People who self‐report a strong moral character can be motivated either to *be* moral or to *appear* moral (Reed & Aquino, 2003; Trivers, 1971). On the one hand, self‐reported moral character may reflect genuine prosocial motives such as compassion (Reed & Aquino, 2003) and guilt proneness (Cohen et al., 2012). Accordingly, it is widely believed that self‐reported moral character predicts more prosocial and less unethical behaviours (Aquino & Reed, 2002; DeCelles et al., 2012; Lee et al., 2008). On the other hand, from an evolutionary perspective, people may strive to maintain a moral reputation because appearing to be a good co‐operator and avoiding social exclusion have long been essential for survival (Nowak & Sigmund, 2005; Sperber & Baumard, 2012; Vonasch et al., 2018). One of the best ways to acquire and maintain a moral reputation is to display, and even internalize, a strong moral character (Heintz et al., 2016; Trivers, 1971). Facing various moral dilemmas in daily life, moral character can facilitate socially desirable reactions in a prompt and heuristic way (Hardy & Van Vugt, 2006; Jordan et al., 2016; Jordan & Rand, 2020), and serve to appear moral to others (Batson et al., 1997, 1999; Everett et al., 2016; Shaw et al., 2014). Therefore, moral character based on self‐reports may have two major components, including not only a ‘genuine’ desire to be moral but also a reputation‐driven desire to appear moral.

Although the influences of reputation management and self‐reported moral character on moral decision making have been discussed a lot, little is known about their joint effects on moral decisions. The relative strength of reputation management motives is likely to vary in different situations (e.g. in public versus in private; Griskevicius et al., 2010) and between individuals. Here, we capture reputation management motives at the individual difference level. People higher on reputation management motives are more attentive to their self‐presentation (Blader & Chen, 2012; Flynn et al., 2006) and their social status (Griskevicius et al., 2010). Especially among individuals who are strongly motivated by reputation management, we suggest an association between self‐reported moral character and moral double standards on the self and others.

### Moral double standards

Serving the goal to ‘appear moral, yet, if possible, avoid the cost of actually being moral’ (Batson et al., 1999, p. 525), moral hypocrisy can manifest as people's moral double standards, that is, being more lenient on themselves as compared to others (Lammers, 2012; Polman & Ruttan, 2012; Valdesolo & DeSteno, 2008; Weiss et al., 2018). To attain or maintain a moral reputation, the ‘reputation managing’ component of self‐reported moral character may play a role, and prompt people to judge others' misdeeds more harshly than their own. First, condemnation of others' misdeeds sends a strong signal about one's virtues to social partners. Previous studies have shown that individuals who morally condemn wrongdoers are viewed as more trustworthy (Everett et al., 2016; Jordan et al., 2017; Simpson et al., 2013). People express moral disgust towards transgressions in order to appear moral (Kupfer & Giner‐Sorolla, 2017); and when participants express outrage towards wrongdoing, their moral self‐concept is enhanced (Rothschild & Keefer, 2017). Even in anonymous settings, people condemn other transgressors harshly as a heuristic to establish a moral reputation (Crockett, 2017; Jordan & Rand, 2020). Thus, people may judge others' misdeeds harshly to ‘appear moral’.

Second, although doing good deeds leads to a good reputation, it often comes with a cost to self‐interest (e.g. time or money; Hardy & Van Vugt, 2006; Jordan et al., 2016). Thus, people whose moral character is based on a self‐interested desire for a moral reputation may justify or downplay the severity of their own transgressions to avoid the cost of actually being moral. Combined, people can condemn others' misdeeds and impose lenient standards on themselves at the same time, that is, show moral double standards. We, therefore, reason that people who self‐report a strong (vs. weak) moral character can show moral double standards as a manifestation of their high (vs. low) reputation management motives.

### The current research

In the current research, three studies examined the relationship between self‐reported moral character and moral double standards, and how the relationship is moderated by the actors' reputation management motives. Moral character can manifest in various forms. While previous studies often focus on one particular form of moral character (e.g. Hertz & Krettenauer, 2016; Schmitt et al., 2005), to test the generalizability of our proposition, we operationalized moral character differently across the three studies, respectively, as Benevolence and Universalism values, justice sensitivity, and moral identity. Consistent with previous studies (e.g. Anderson et al., 2015; Flynn et al., 2006; Griskevicius et al., 2010), we operationalized reputation management motives as individual differences in motives to attain social esteem and status. More specifically, reputation management motives were measured with people's self‐importance of Power and Achievement values, self‐monitoring of socially desirable behaviours, and their concern for social status. Moreover, the predictions on moral double standards were tested through various methods, including large‐scale multination panel data (Study 1), vignettes (Study 2), and a behavioural experiment (Study 3). As such, the present research sought to establish converging evidence for our propositions across multiple conceptualizations of moral character and reputation management motives, while using complementary research methods. We, therefore, describe our propositions and findings using the terms ‘moral character’ and ‘reputation management motives’ across the three studies. We summarized different measures of these two individual difference variables in Table 1, and elaborated on the measures in the respective studies. We expect a three‐way interaction effect among moral character, reputation management motives, and moral target, such that self‐reported moral character predicts stronger moral condemnation of transgressions committed by others than oneself, which is especially true for people with strong (vs. weak) reputation management motives.

**TABLE 1:** Measures of moral character and reputation management motives in Studies 1 to 3

	Moral character	Reputation management motives
Study 1	Benevolence and Universalism values (Schwartz, 2012)	Power and Achievement values (Schwartz, 2012)
Study 2	Justice sensitivity (Schmitt et al., 2005)	Self‐monitoring (Snyder, 1974)
Study 3	Moral identity (Aquino & Reed, 2002)	Concern about status (Blader & Chen, 2011)

Study 1 used existing archived data; Studies 2 and 3 determined the sample size before data collection and analysis, and did not collect more data after data analysis. A‐priori power analyses were conducted with G*Power (Version 3.1; Faul et al., 2009) where applicable. All measures, manipulations, and exclusions (if any) were disclosed in the respective studies. The panel data and codebook for Study 1 can be downloaded at https://www.europeansocialsurvey.org/data/download.html?r=2; we only provided our code for analyses. The data, analyses, codebook, and experimental materials for the other studies have been uploaded on the Open Science Framework, and can be accessed at https://osf.io/3f8br/ (Dong, 2021).

## STUDY 1

Study 1 examined our hypotheses with a multinational representative sample. We employed data from the second‐round European Social Survey (ESS, 2004). Different from other waves, the 2004‐wave ESS included a unique module about economic morality, which recorded more than 40,000 respondents' self‐reported frequencies of normative transgressions and their judgements of similar transgressions. Although neither of them directly measured moral condemnations of own or others' transgressions, we used self‐reported frequencies of transgressions as a proxy of moral standards on the self and judgements of transgressions without specific targets as a proxy of moral standards on others. The panel data captured people's moral character and reputation management motives with their personal identifications with relevant values (i.e. Benevolence and Universalism, versus Power and Achievement, respectively). Values are ‘guiding principles in life’ (Schwartz, 2012, p. 16), which predict a variety of moral and prosocial attitudes and behaviours (Schwartz, 2010). Based on our line of reasoning, we expected a stronger correlation of moral character with judgements of transgressions than with frequencies of own transgressions when people had stronger (vs. weaker) reputation management motives.

### Method

#### Participants

The 2004‐wave ESS collected data of 47,537 participants from 25 countries via face‐to‐face interviews. The current research included 34,323 participants (16,016 males, 18,254 females, and 53 no answer; *M*_age_ = 46.24, *SD* = 17.86), who gave valid answers to all the examined questions. Put differently, we excluded respondents whose answers were recorded as ‘Refusal’ ‘Do not know’ or ‘No answer’ on any of the targeted questions. Most included participants self‐identified as belonging to an ethnic majority group in their country (i.e. 32,462 people; 94.6% ethnic majority vs. 3.7% ethnic minority vs. 1.7% other).

#### Measures

All respondents answered items in a fixed order (see Supplementary Materials for the items in the order they were measured). They first indicated the moral wrongness of four different transgressions (*α* = .71; e.g. ‘How wrong, if at all, is someone making exaggerated/false insurance claim?’ on a 4‐point scale from 1 = *Not wrong at all* to 4 = *Seriously wrong*). After responding to some unrelated questions, participants then indicated their own transgressive frequencies in seven different scenarios (ordinal *α* = .84; e.g. ‘How often, if ever, have you made an exaggeration or false insurance claim in the last five years?’ on a scale with 1 = *Never*, 2 = *Once*, 3 = *Twice*, 4 = 3 to 4 times, and 5 = *5 times or more*).

European Social Survey captured moral character and reputation management motives with the Schwartz Value Survey (Schwartz, 2012), in which the motivational goals ‘self‐transcendence’ and ‘self‐enhancement’ largely overlap with the two concerned constructs, respectively. As moral character, the five items within the ‘self‐transcendence’ goal were all included (*α* = .73), representing the values of Benevolence (two items; e.g. ‘Important to help people and care for others well‐being’) or Universalism (three items; e.g. ‘Important to understand different people’). As reputation management motives, four out of six items within the ‘self‐enhancement’ goal were included (*α* = .72), representing the values of Power (two items; e.g. ‘Important to get respect from others’) and Achievement (two items; e.g. ‘Important to show abilities and be admired’)—but not Hedonism (two items; e.g. ‘Important to seek fun and things that give pleasure’; which does not imply reputation‐related values). Participants were asked to indicate the extent to which a hypothetical person with the aforementioned values was like themselves (on a 6‐point scale; 1 = *Very much like me*, 6 = *Not like me at all*). We reverse‐coded the scores for ease of comprehension, and averaged the items under the respective constructs.

### Results

We conducted separate analyses for averaged frequencies (*Mdn* = 1.00 meaning ‘*Never*’) of own transgressions and perceived wrongness of transgressions (*M* = 3.27, *SD* = 0.51) due to their different data types and the highly skewed distribution of own transgressive frequencies (skewness = 2.58; see Figure S1).

**FIGURE 1:**
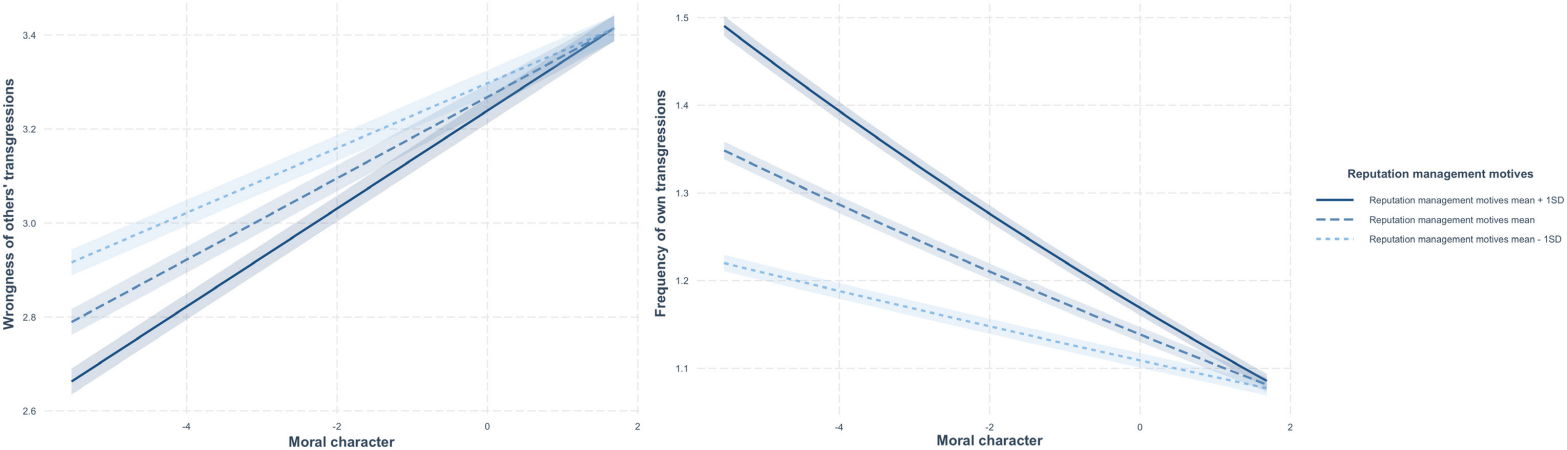
Perceived wrongness of others' transgressions (left panel) and self‐reported frequency of own transgressions (right panel) as a function of moral character (as benevolence and universalism values) and reputation management motives (as power and achievement values) in study 1 (*N* = 34,323).

We first performed a two‐level linear mixed model, examining perceived wrongness of transgressions as a function of moral character (*M* = 4.83, *SD* = 0.69) and reputation management motives (*M* = 3.56, *SD* = 0.99; both grand mean‐centred after averaging the value items), as well as their two‐way interaction, with random intercepts for participant id and transgressive scenario, and participant id nested within country (Table S1). We found a positive correlation of self‐reported moral character (*β* = .11, *p* < .001, 95% CI [0.101, 0.115]), but a negative correlation of reputation management motives (*β* = −.04, *p* < .001, 95% CI [−0.043, −0.030]), with perceived wrongness of transgressions. Crucially, a significant two‐way interaction (*β* = .02, *p* < .001, 95% CI [0.016, 0.028]) revealed that moral character was associated with harsher judgement of transgressions, for people with stronger (+1 *SD*; *B* = .10, *SE* = .004, *t* = 26.09, *p* < .001, $\eta_{p}^{2}$ = .019) more so than weaker (−1 *SD*; *B* = .07, *SE* = .004, *t* = 19.50, *p* < .001, $\eta_{p}^{2}$ = .011) reputation management motives (Figure 1). The findings supported our reasoning, showing a stronger link between moral character and moral harshness towards others who were strongly (vs. weakly) motivated by reputation.

We then fitted a two‐level Poisson generalized linear mixed model (GLMM) on people's self‐reported frequency of transgressions, again with grand mean‐centred moral character, reputation management motives, and their interaction effect as predictors, including random intercepts for participant id and transgressive scenario, and participant id nested within country (Table S1). Moral character negatively (*β* = −.03, *p* < .001, 95% CI [−0.034, −0.027]), and reputation management motives positively (*β* = .03, *p* < .001, 95% CI [0.023, 0.030]), correlated with the frequency of people's own economic transgressions. Moreover, we found a significant two‐way interaction (*β* = −.01, *p* < .001, 95% CI [−0.017, −0.010]). Moral character was related to fewer self‐reported transgressions, especially for people with higher (*B* = −.04, *SE* = .003, *z* = −15.96, *p* < .001, $\eta_{p}^{2}$ = .010) rather than lower (*B* = .08, *SE* = .004, *z* = −12.47, *p* < .001, $\eta_{p}^{2}$ = .005) reputation management motives (Figure 1). To demonstrate the robustness of our findings, a binomial GLMM (after transforming transgressive frequency into a binary variable representing none [= 0] or any [= 1] transgression) and another Poisson GLMM (after log‐transforming the skewed transgressive frequency; skewness = 1.69) were explored and supported our main findings (Table S2).

### Discussion

Study 1 examined daily‐life economic transgressions in a multination representative dataset. As in many previous studies, we found positive correlations of moral character (as Benevolence and Universalism values) with harsher judgements of moral wrongness and fewer self‐reported transgressions (Aquino & Reed, 2002; Hertz & Krettenauer, 2016). Somewhat surprisingly, the main effect of reputation management motives (as Power and Achievement values) predicted lenient moral judgements and more self‐reported transgressions. Nonetheless, the interaction between moral character and reputation management was in the predicted direction.

More specifically, there was a stronger correlation between moral character and moral harshness in general moral judgements for people who were strongly (vs. weakly) motivated by reputation. A similar pattern also emerged in self‐reported frequency of transgressions, such that people having higher moral character reported fewer own transgressions especially when they were high (vs. low) on reputation management motives. This may have been because self‐reported frequency of transgressions, especially in face‐to‐face interviews, reflected more of strategic self‐presentation, not only actual moral standards on the self (Dong et al., 2019; Shaw et al., 2014). This explanation is conceptually consistent with our reasoning, in that people reporting high (vs. low) moral character and reputation management motives not only imposed harsher standards on others but also downplayed their own transgressions to serve moral reputation. The results on self‐reported transgressions should be interpreted with caution, however, because it is not clear whether (1) people transgressed and reported the actual frequency, or (2) people transgressed but underreported the actual frequency to serve a positive reputation—especially for those who were high (vs. low) on both moral character and reputation management motives.

## STUDY 2

While Study 1 provided preliminary evidence for our line of reasoning in a high‐powered multination study, it also had two major limitations. First, self‐reported transgressive frequency versus perceived wrongness of transgressions without specific targets were not direct measures of people's moral standards on the self versus others. Relatedly, these two measures were not captured on the same scale and were not directly compared. Separate analyses on the two measures thus did not account for the covariance induced by identical respondents (who answered both questions about self and others). Second, the content of transgressions was not perfectly aligned between measures referring to self and other. To address these limitations, we sought additional evidence showing that moral character and reputation concerns drive different judgements for self versus others for *identical* transgressions. Using vignettes with more rigorous experimental controls, Study 2 tested moral double standards with consistent transgressions and measures of moral blame in the self and other conditions. For identical transgressions, we expected a three‐way interaction effect among moral character, reputation management motives, and moral target (i.e. self versus others), such that self‐reported moral character relates to moral harshness towards others more than oneself, especially among people who are strongly motivated by reputation management.

In Study 2, we operationalized reputation management motives as self‐monitoring—the propensity to modify behaviours depending on anticipated social approval (Flynn et al., 2006; Snyder, 1974), and moral character as dispositional justice sensitivity (Schmitt et al., 2005). In social interactions with others, self‐reported justice sensitivity strongly correlates with other‐related concerns (e.g. role taking, empathy, and social responsibility; Schmitt et al., 2005). People who report being highly sensitive to (in)justice make harsher moral judgements of others' harm‐related behaviours (Yoder & Decety, 2014), and are more likely to cooperate and distribute resources fairly in economic game studies (Fetchenhauer & Huang, 2004). In summary, sensitivity to injustice is associated with high moral standards, and justice sensitivity is often regarded as a central component of moral character (Kohlberg, 1976).

### Method

#### Participants

An average effect size of social psychological studies ($\eta_{p}^{2}$ = .04; Richard et al., 2003) required a sample size of *N* = 191, to detect our intended moral character by reputation management motives by moral target interaction effect with 80% power at an alpha level of 0.05. We recruited 198 participants from the Netherlands (46 males and 152 females; *M*_age_ = 22.29, *SD* = 3.84; 80.3% as students) to complete our survey on SurveySwap, which is an online platform where Dutch students often publish and complete surveys as an exchange. We did not exclude any participants from analyses.

#### Design and materials

Participants were randomly assigned to either a self‐as‐target (*n* = 102) or other‐as‐target (*n* = 96) condition. In both conditions, participants followed the same procedure: They first evaluated four transgression scenarios in a randomized order (see the SM; adapted from Lammers, 2012; Weiss et al., 2018). They then completed the measures of justice sensitivity and self‐monitoring. The two measures were presented in a counterbalanced order with specific items in a fixed order.

In the self‐as‐target condition, we described four organizational scenarios and asked participants to imagine themselves enacting questionable behaviours in these scenarios (e.g. sharing information about a confidential project with a friend). After reading each scenario, participants answered four questions about moral blame on a 7‐point scale ranging from 1 = *not at all* to 7 = *absolutely* (*α* = .62 across 16 items; e.g. ‘It would reflect poorly on me if I shared information about this confidential project with my friend’, as in Weiss et al., 2018). In the other‐as‐target condition, participants read about their ‘co‐worker’ committing identical transgressions and indicated their judgements on equivalent items (*α* = .70 across 16 items).

We then measured people's moral character with their justice sensitivity. The original scale comprised three dimensions of perpetrator sensitivity (benefiting from unjust events), observer sensitivity (observing others being treated unjustly), and victim sensitivity (experiencing injustice towards oneself). We only measured the first two dimensions given their particular relevance for people's moral character. Victim sensitivity reflects moral concerns but in a self‐protective and even egoistic way. As such, people high on victim sensitivity were more likely to make unfair offers, whereas sensitivity to justice from both the perpetrator and observer perspectives correlated positively with fair decisions (Fetchenhauer & Huang, 2004). Twenty questions were rated on a 7‐point scale ranging from 1 = *Not at all* to 7 = *Exactly*, including 10 on perpetrator sensitivity (*α* = .89; e.g. ‘It worries me for a long time when others have to fix my carelessness’) and 10 on observer sensitivity (*α* = .81; e.g. ‘I am upset when someone does not get a reward he/she has earned’). The 20 items aggregated yielded satisfactory internal consistency (*α* = .90) and a strong correlation between the two dimensions (*r* = 0.60). We, therefore, aggregated the two dimensions as an overall indicator of justice sensitivity. However, it is still controversial whether perpetrator sensitivity and observer sensitivity should be separated or integrated in analyses. Though perpetrator perspective seems to relate more with the self‐as‐target condition, and observer perspective corresponds to the other‐as‐target condition, these two dimensions both predict other‐related concerns and behaviours (Fetchenhauer & Huang, 2004; Schmitt et al., 2005). We, therefore, performed alternative analyses with the mean scores of, respectively, perpetrator or observer sensitivity items as independent indicators of moral character in the SM.

We measured participants' reputation management motives with their total score on the self‐monitoring scale (ordinal *α* = .70 across 25 binary items answered with ‘Yes’ or ‘No’; Snyder, 1974). However, though the original self‐monitoring scale was developed as a single dimension measure (Snyder, 1974), some follow‐up studies demonstrated that self‐monitoring was better captured as a three‐dimension construct including acting (e.g. ‘I can make impromptu speeches on topics about which I have almost no information’), extraversion (e.g. ‘In a group of people I am rarely the centre of attention’ [reverse‐coded]), and other‐directness (e.g. ‘I guess I put on a show to impress or entertain people’; Briggs et al., 1980). Among the three dimensions, other‐directness may be the best reflection of reputation management motives, as it depicts people's willingness to change their behaviours to win others' favour (Briggs et al., 1980). We, therefore, performed the alternative analyses (see the SM) with the 10‐item other‐directness subscale (ordinal *α* = .77) rather than the 25‐item self‐monitoring scale representing reputation management motives in the SM. The alternative analyses showed a consistent pattern of results and largely substantiated our main findings.

### Results

We performed a two‐level linear mixed model, regressing moral blame (*M* = 4.54, *SD* = 1.89) on target (self = −1, other = 1), mean‐centred moral character (*M* = 3.15, *SD* = 0.89), reputation management motives (*M* = 12.08, *SD* = 3.67), and their two‐way and three‐way interactions, with random intercepts for participant id, transgressive scenario, and moral blame item (Table S3). We found a significant interaction between moral character and reputation management motives (*β* = −.20, *p* < .001, 95% CI [−0.29, −0.12]). Moral character positively correlated with overall moral blame, only for people with weaker (−1 *SD*; *B* = .60, *SE* = .14, *t* = 4.36, *p* < .001, $\eta_{p}^{2}$ = .08) but not stronger (+1 *SD*; *B* = −.25, *SE* = .11, *t* = −2.20, *p* = .029, $\eta_{p}^{2}$ = .02) reputation management motives. More importantly, the hypothesized three‐way interaction was significant (*β* = .27, *p* < .001, 95% CI [0.14, 0.40]).

**FIGURE 2:**
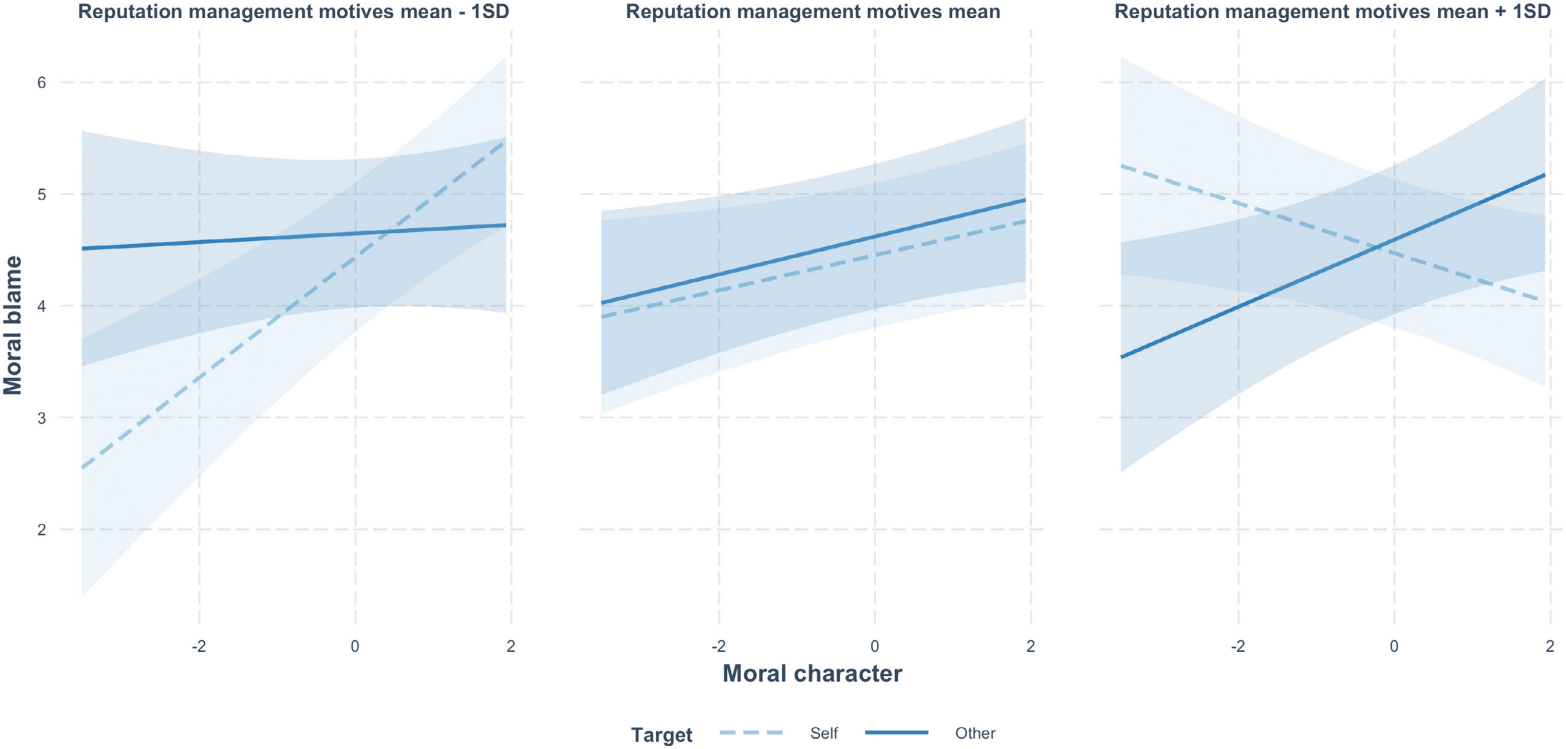
Moral blame of own versus others' hypothetical transgressions, as a function of moral character (as justice sensitivity) and reputation management motives (as self‐monitoring) in study 2 (*N* = 198).

As shown in Figure 2, moral character interacted with moral target, more for people who had high (+1 *SD*; *B* = −2.40, *SE* = .60, *t* = −4.02, *p* < .001, $\eta_{p}^{2}$ = .32) than low (−1 *SD*; *B* = −1.27, *SE* = .34, *t* = −3.77, *p* < .001, $\eta_{p}^{2}$ = .29) reputation management motives. When reputation management motives were low (−1 *SD*), moral character predicted a stronger blame for the self (*B* = .40, *SE* = .17, *t* = 2.33, *p* = .021, $\eta_{p}^{2}$ = .11) but not for others (*B* = .18, *SE* = .12, *t* = 1.49, *p* = .138, $\eta_{p}^{2}$ = 0.04). When reputation management motives were high (+1 *SD*), instead, moral character was positively associated with moral blame for others (*B* = .26, *SE* = .12, *t* = 2.17, *p* = .031, $\eta_{p}^{2}$ = 0.10) but negatively associated with moral blame for the self (*B* = −.04, *SE* = .14, *t* = 0.30, *p* = .767, $\eta_{p}^{2}$ = .002).

### Discussion

Different from Study 1 where people indicated both perception and enactment of civic transgressions, Study 2 employed a between‐subjects design where people either evaluated themselves or others. With student samples and hypothetical organizational transgressions, Study 2 extended the Study 1 findings and showed a significant relation between moral character (as justice sensitivity) and moral double standards among people with high but not low reputation management motives (as self‐monitoring). For those who self‐reported both high (vs. low) moral character and reputation management motives, people did not only show more harshness towards others (as in Study 1) but also more leniency towards themselves.

## STUDY 3

In Study 3, we conducted a pre‐registered behavioural experiment where people evaluated identical (im)moral behaviours in the self and other conditions. The pre‐registration can be accessed on Open Science Framework (https://osf.io/75wrm/?view_only=48e6d0fe93274c319faa8d72705f6bf5). In the self condition, the experimental task examined people's actual (un)fair choices in resource distribution, and subsequent evaluations of their own choices. As in Studies 1 and 2, we predicted a relation between self‐reported moral character and moral double standards moderated by reputation management motives. More specifically, people high (vs. low) on self‐reported moral character should judge unfair decisions as unacceptable when made by others but not themselves, and this moral double standard should be more pronounced when people are strongly (vs. weakly) motivated by reputation.

In this study, we captured moral character with one of its most widely used measures in studies of moral judgement and behaviour—*moral identity* (Aquino & Reed, 2002; Hertz & Krettenauer, 2016). Moral identity can be defined as the centrality of being moral to one's identity. In other words, the more people feel that being caring, compassionate, fair, friendly, generous, helpful, hardworking, honest, and kind are central for defining their personal identity, the higher their moral identity (Aquino & Reed, 2002). Moral identity is associated with strong moral awareness and less selfish behaviours even when people experience the freedom to pursue self‐interest (DeCelles et al., 2012). As a measure of reputation management motives, we asked people to indicate their concern about status. Status often builds on social prestige and esteem, such that status seekers are more motivated to cultivate a good reputation than average others (Blader & Chen, 2011; Flynn et al., 2006; Griskevicius et al., 2010). We reported a Pilot Study in the SM that we conducted prior to Study 3, using identical measures of moral character and reputation management motives while combining third‐party punishment options with judgements of others' behaviours. We will discuss its main findings together with the Study 3 findings.

### Method

#### Participants

An a‐priori power analysis suggested a sample of *N* = 191 to detect the hypothesized three‐way interaction effect ($\eta_{p}^{2}$ = .04 as in the Pilot Study in the SM) with 80% at an alpha level of 0.05. We estimated that only half of the participants in the self condition would make a selfish rather than a fair choice (as in the Pilot Study in the SM; similarly in Dong et al., 2019); we, therefore, intended 300 participants (i.e. *n* = 200 in the self and *n* = 100 in the other condition). We had 301 participants (158 males, 142 females, and 1 other; *M*_age_ = 41.6 years, *SD* = 12.9) from the United States recruited on the crowdsourcing platform TurkPrime.com (Peer et al., 2017). Most participants self‐identified as ‘White’ (80.4%) or ‘Black or African American’ (13.0%). All participants were included in further analyses.

#### Design and procedure

Following basic demographic questions, participants indicated their moral character and reputation management motives in a randomized order. The moral character measure was the 5‐item internalization subscale of the Self‐Importance of Moral Identity Questionnaire (Aquino & Reed, 2002). Participants were asked to read nine adjectives (e.g. generous, fair) and rated self‐importance of these characteristics (*α* = .78; e.g. ‘It would make me feel good to have these characteristics’, on a 7‐point scale ranging from 1 = *Strongly disagree* to 7 = *Strongly agree*). We adopted a validated measure of concern about social esteem and status to measure reputation management motives (*α* = .94 across 10 items; e.g. ‘I find it important that others acknowledge my status’ ‘I wish to have high status’, as in Blader & Chen, 2011, 2012). The order of specific items in each measure did not vary between participants.

Participants were subsequently enrolled in an ostensibly unrelated interaction game, with three roles: Distributor, Recipient, and Observer (see the SM for detailed instructions). Participants were randomly assigned to be Distributors (*n* = 201) or Observers (*n* = 100). They made incentivized decisions with a 5% chance to win an actual bonus as indicated in the game. Since Recipients do not make any active decisions, we did not recruit new participants to play this role but sent the corresponding bonus to people who participated in one of our previous studies. We first explained that Distributors should assign a $10 bonus between the self and another online Recipient, with two choices: (1) keeping $8 and giving $2 to the Recipient or (2) keeping $5 and giving $5 to the Recipient. We then explained that Observers would know the choice of the same‐group Distributor, receive a fixed $3 bonus, and cannot influence the payoffs of the Distributor and the Recipient. Participants were then randomly assigned to their role of a Distributor or an Observer. After making a Distributor choice, Distributors indicated ‘How acceptable do you think your choice is in the game?’ (on a 7‐point scale ranging from to −3 = *Completely unacceptable* to 3 = *Completely acceptable*). In the Observer role, participants reviewed both selfish and fair choices in a randomized order. For each possible choice, they answered the question ‘How acceptable do you think the Distributor's choice is in the game?’ based on the same scale.

### Results

Among the included Distributors (*N* = 201), 69 (34.3%) participants made an $8/$2 offer and 132 (65.7%) participants made a $5/$5 offer. We first analysed Distributors' actual behaviour, and then contrasted their judgement of own behaviour with Observers' judgement of identical behaviour, both as reflections of people's moral standards for themselves.

#### Moral behaviour

We conducted a binary logistic regression to examine the roles of mean‐centred moral identity (*M* = 6.00, *SD* = 0.92) and reputation management motives (*M* = 4.96, *SD* = 1.39), and their interaction, in Distributors' choice between $8/$2 (= 0) and $5/$5 (= 1). Moral identity predicted a higher likelihood to choose $5/$5 rather than $8/$2 (*z* = 2.12, *p* = .034). Neither reputation management motives (*z* = 1.80, *p* = .071) nor the two‐way interaction (*z* = −1.25, *p* = .212) correlated with a fair rather than a selfish choice.

#### Moral judgement

We contrasted judgements of a $8/$2 offer in a linear regression, as a function of moral character, reputation management motives, and moral target (i.e. Observers judging others whilst Distributors evaluating themselves). We found significant main effects of moral character (*β* = −.28, *p* = .004, 95% CI [−0.42, −0.13]) and moral target (*β* = −.47, *p* = .002, 95% CI [−0.36, −0.07]), suggesting a negative relation between moral character and moral leniency, as well as more lenient judgements of own than others' selfish distribution. A two‐way interaction between moral character and moral target also emerged (*β* = −.23, *p* < .001, 95% CI [−0.37, −0.09]), such that moral character predicted moral harshness towards others (*B* = −.87, *SE* = .18, *t* = −4.82, *p* < .001, $\eta_{p}^{2}$ = .12) but not towards the self (*B* = .01, *SE* = .20, *t* = 0.03, *p* = .979, $\eta_{p}^{2}$ < .001). Crucially, as predicted, there was a significant three‐way interaction effect (*β* = −.19, *p* = .010, 95% CI [−0.33, −0.04]).

**FIGURE 3:**
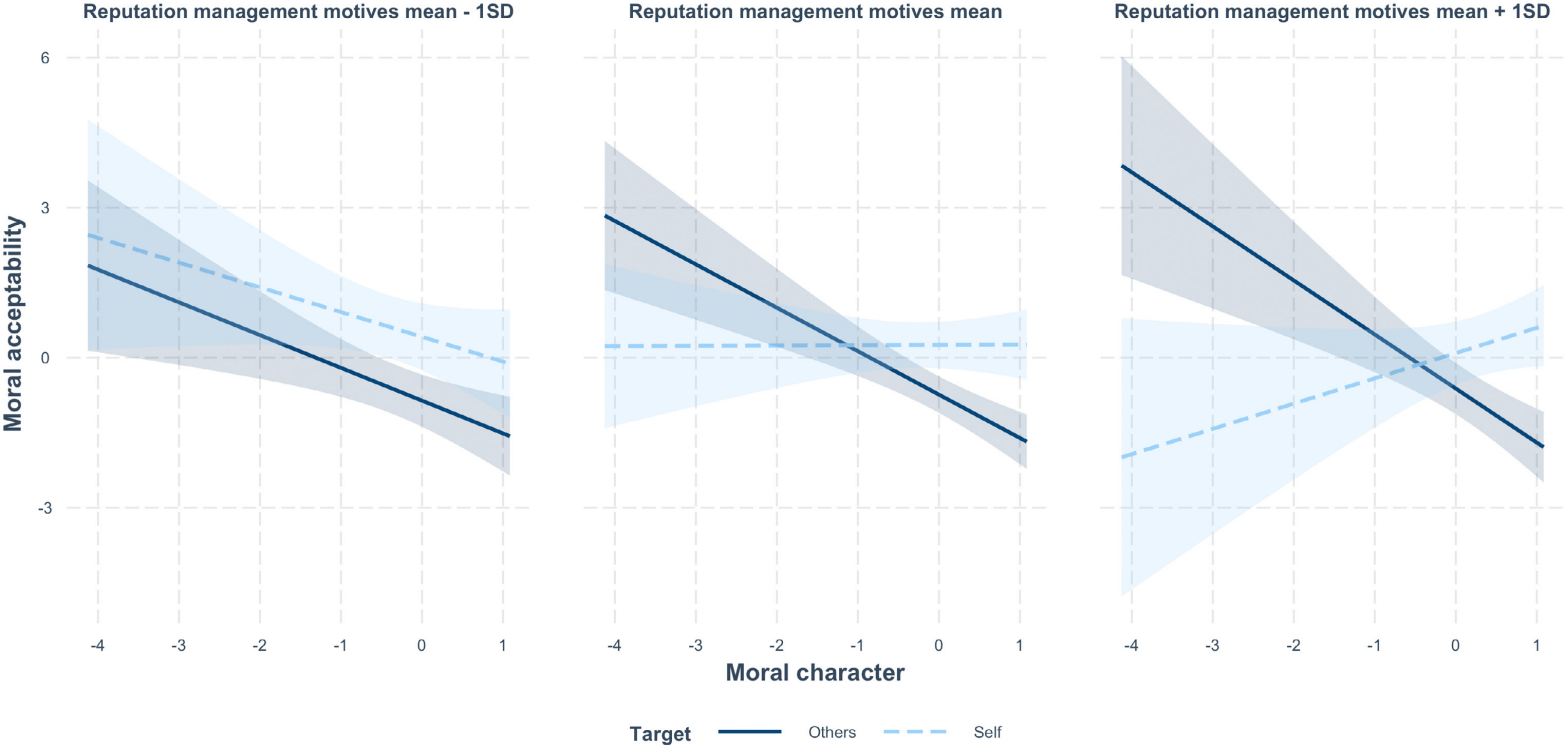
Moral acceptability judgements of own and others' selfish choice in an actual dictator game, as a function of moral character (as moral identity) and reputation management motives (as concern about status) in study 3 (*N* = 169).

Further simple slope analyses revealed that the two‐way interaction between moral character and moral target only emerged among people with higher (+1 *SD*; *B* = −.83, *SE* = .21, *t* = −3.90, *p* < .001, $\eta_{p}^{2}$ = .09) but not lower (−1 *SD*; *B* = −.15, *SE* = .17, *t* = −0.90, *p* = .37, $\eta_{p}^{2}$ = .01) reputation management motives. As shown in Figure 3, self‐reported moral character predicted harsher judgements of others' selfish acts among people who were more (+1 *SD*; *B* = −1.08, *SE* = .25, *t* = −4.28, *p* < .001, $\eta_{p}^{2}$ = .10) rather than less (−1 *SD*; *B* = −.66, *SE* = .22, *t* = −3.02, *p* = .003, $\eta_{p}^{2}$ = .05) motivated by reputation.

In comparison, in moral judgements of a $5/$5 offer, we only found a significant relation between moral character and moral judgement (*β* = −.14, *p* = .045, 95% CI [−0.27, −0.01]). None of the other correlations were significant (*p*s >.346). In the SM, we report a Pilot Study (*N* = 201; 117 males and 84 females; *M*_age_ = 37.9 years, *SD* = 10.5), which was based on a third‐party punishment game and was conducted prior to Study 3. In the Pilot Study, Observers had an additional opportunity to punish Distributors with their own endowment, and Distributors were made aware of such punishment. The significant three‐way interaction of moral character, reputation management motives, and moral target on judgements of a $8/$2 selfish offer replicated. Together, these findings provide solid evidence in support of our hypotheses, suggesting that in judgements of selfish or immoral acts, the relation between moral character and differential standards on the self versus others were contingent on reputation management motives.

### Discussion

In an actual behavioural experiment with a selfish versus a fair choice, we found positive relations of moral character (as moral identity) with not only harsher subjective judgements but also more moral behaviours. However, as in Studies 1 and 2, the harsher moral judgements were mainly manifested in evaluations of others but not oneself, and were especially true among reputation‐motivated individuals (i.e. who were concerned about status).

Moreover, individuals with both strong moral character and reputation management motives only condemned others' selfishness more harshly but did not appraise others' fairness more positively (in both Study 3 and its Pilot Study in the SM). This finding does not necessarily mean that people receive no credit for approving of others' good deeds, but at least suggests that people who self‐report a strong moral character are more prone to manage their reputation through moral condemnation rather than approval. One possible reason is that compared to praising others, condemning others is a more costly signal because it risks retaliation, and costly signals tend to be received as more authentic than cheap signals (Jordan et al., 2017). People may thus readily employ condemnation as a strategy to demonstrate their righteousness to others.

It should also be noted that Study 3 examined moral character as the internalization—instead of the symbolization—subscale of the moral identity measure. Whereas the symbolization subscale more directly reflects the self‐presentation motive (e.g. ‘I often wear clothes that identify me as having these characteristics’; Aquino & Reed, 2002), previous research typically regards the internationalization subscale as reflecting a ‘genuine’ moral character. Our findings then suggest that many self‐reported measures—even though widely believed as reflecting a ‘genuine’ moral character—can have the ‘reputation managing’ component and relate to moral double standards.

## GENERAL DISCUSSION

A well‐known Golden Rule of morality is to treat others as you wish to be treated yourself (Singer, 1963). People with a strong moral character might be expected to follow this Golden Rule, and judge others no more harshly than they judge themselves. However, when moral character is measured by self‐reports, it is often intertwined with socially desirable responding and reputation management motives (Anglim et al., 2017; Hertz & Krettenauer, 2016; Reed & Aquino, 2003). The current research examines the potential downstream effects of moral character and reputation management motives on moral decisions. By attempting to differentiate the ‘genuine’ and ‘reputation managing’ components of self‐reported moral character, we posited an association between moral character and moral double standards on the self and others. Imposing harsh moral standards on oneself often comes with a cost to self‐interest; to signal one's moral character, criticizing others' transgressions can be a relatively cost‐effective approach (Jordan et al., 2017; Kupfer & Giner‐Sorolla, 2017; Simpson et al., 2013). To the extent that the demonstration of a strong moral character is driven by reputation management motives, we, therefore, predicted that it would be related to increased hypocrisy, that is, harsher judgements of others' transgressions but not stricter standards for own misdeeds.

Across four studies varying from civic transgressions (Study 1), organizational misconducts (Study 2), to selfish decisions in economic games (Study 3 and the Pilot Study in the SM), we found consistent evidence that people reporting a strong (vs. weak) moral character were more likely to judge others' misdeeds harshly, especially for those highly motivated by reputation. This amplified moral harshness towards others was sometimes also accompanied with increased moral leniency towards the self (Study 3 and the Pilot Study in the SM). Taken together, self‐reported moral character relates to differential moral standards on the self versus others, which was especially true for reputation‐motivated individuals.

Although Study 1 only provided circumstantial evidence by interpreting moral judgements without specific targets and self‐reported transgressive frequencies as a proxy of the ‘reputation managing’ component of self‐reported moral character, we have good reasons to believe that these interpretations are legitimate. First, people often apply general moral rules to judgements of others instead of themselves (Dong et al., 2021). Second, self‐reported moral performance is often influenced by strategic self‐presentation (Dong et al., 2019; Shaw et al., 2014). As shown in our studies, people high (vs. low) on moral character reported fewer own transgressions (Study 1) when highly (vs. weakly) motivated by reputation management. However, they did not act more or less selfishly (Study 3).

Furthermore, Studies 2 and 3 consolidated our proposition by showing a significant interaction between moral character and target of moral judgements (i.e. self vs. other), only for people with high but not low reputation management motives. These findings were replicated across a variety of individual difference measures of moral character (including Benevolence and Universalism values, justice sensitivity, and moral identity) and reputation management motives (including Power and Achievement values, self‐monitoring of socially desirable behaviours, and concern about social esteem and status), and emerged only when moral judgements had a salient influence on people's reputation (e.g. when the appraised behaviour was unfavourable rather than favourable in Study 3).

### Theoretical contributions

The current findings contribute to the literature on both moral character and reputation management. Previous theorizing generally implies that moral character is genuinely and unconditionally good (Aquino & Reed, 2002; Kamtekar, 2004; Walker et al., 1987; Walker & Frimer, 2007). Consistent with this ‘genuine’ perspective on moral character, we found positive correlations of moral character with stringent moral judgements (Studies 1 and 3) and a high likelihood to behave morally (Study 3), although the relation between inherent moral character and actual moral deeds may be obscured by the presence of external sanctions (e.g. third‐party punishment in the Pilot Study in the SM). More importantly, we complement previous studies on moral character by making two novel contributions.

First, the present studies suggest that there are both ‘genuine’ and ‘reputation managing’ components of self‐reported moral character. Although this idea was implied in many previous studies (e.g. Anglim et al., 2017; Brick et al., 2017; Dong et al., 2019; Hertz & Krettenauer, 2016; Shaw et al., 2014), our work empirically demonstrates that people who report a strong moral character can be sensitive to moral contexts, and strategically tailor their moral performances accordingly. In particular, people may apply flexible moral standards consistent with reputation management goals, and display more moral harshness towards others than towards themselves. The findings accord with perspectives that emphasize the prominent role of reputation management in moral psychology (e.g. Jordan et al., 2016; Vonasch et al., 2018), including phenomena such as moral licencing (Blanken et al., 2015) and moral contagion (Kupfer & Giner‐Sorolla, 2021).

Second, our work illuminates how exactly reputation management motives moderate the link between self‐reported moral character and moral decisions. Beyond previous research suggesting to control for, or eliminate, reputation concerns in moral character measurements (Lee et al., 2008; Paulhus, 1984), these studies demonstrated when, and for whom, moral character precisely predicts moral decisions. When individuals had low reputation management motives, their moral character predicted moral judgements of their own more than others' misdeeds; in contrast, when people were highly motivated to gain a good reputation, moral character only predicted their moral harshness towards others but failed to predict moral decisions for themselves (Study 3 and the Pilot Study in the SM). With the increase of reputation management motives, people who reported a strong (vs. weak) moral character either showed increased hypocrisy by judging others more harshly than themselves (Studies 2 and 3), or showed reduced ‘hypercrisy’ (Lammers, 2012) by judging themselves less harshly than others (the Pilot Study in the SM). Although the specific manifestations of moral double standards varied from moral harshness towards others to moral leniency towards oneself, or both, our findings add more insight to discussions about the effectiveness of moral character measures, by suggesting the importance of taking into account reputation management motives and moral target (e.g. self or others).

### Limitations and future directions

We employed diverse samples and methods to test the reputation management account of moral character; however, at least two important limitations should be noted, respectively, related to the self‐reported nature of our moral character and reputation management motives measures.

First, although our findings showed a positive relationship between moral character and moral double standards, we may not fully differentiate the ‘genuine’ and ‘reputation managing’ parts of self‐reported moral character. People may also internalize reputation management as an integral part of ‘genuine’ moral character.^1^ In this case, moral character can facilitate socially desirable reactions in a prompt and heuristic way, and better serve the goal to appear moral to others (Everett et al., 2016; Hardy & Van Vugt, 2006; Jordan et al., 2016; Jordan & Rand, 2020). This theorizing implies that self‐reported moral character can be strongly and positively correlated with reputation management motives. However, the hypothesized interaction effect between moral character and reputation management motives on moral double standards replicated, regardless of their different correlations across studies (positive and significant in Studies 1 and 3, non‐significant in Study 2, and negative and significant in the Pilot Study in the SM; see Table S6 for specifics). To more formally differentiate the roles of actual and postured moral character in behavioural hypocrisy, future research may integrate self‐ with other‐reports of moral character.

Second, we examined reputation management motives as an individual difference variable, and did not manipulate reputation incentives to show its causal effects. As such, self‐reported reputation management motives could be influenced by concerns about social approval. For example, some research suggests that people may under‐report their actual reputation management motives because pursuing good reputation and high status can be stigmatized (Kim & Pettit, 2015). People may either over‐ or under‐report their reputation management motives, depending on their perception of the motives as socially approved or disapproved.

Relatedly, our findings do not directly elucidate whether people who display moral double standards (1) genuinely believe such behaviours as morally acceptable, or (2) consciously use them as a reputation management strategy. For example, although high moral character and reputation management motives were associated with stringent moral standards on others across our studies, their relation with lenient moral standards on the self seemed to only apply to moral judgements but not to actual behaviours (Study 3 and its Pilot Study in the SM). The extent to which self‐reported moral behaviours reflected actual behaviours or its strategic self‐presentation was also unverifiable (Study 1). However, comparisons between different studies may provide tentative evidence on people's conscious and strategic display of moral double standards as a reputation management strategy. People who self‐reported high (vs. low) moral character and reputation management motives judged themselves more leniently only in relatively anonymous settings (Study 2) but no more leniently with the presence of a third‐party interviewer (Study 1) or observer (Study 3 and its Pilot Study in the SM). Future research may explore the mechanisms of moral double standards in different reputation contexts, and examine moral character and reputation management motives as antecedents to behavioural forms of moral hypocrisy (e.g. saying one thing and doing another; Dong et al., 2019; Effron et al., 2018).

## CONCLUSION

How moral character guides moral judgements and behaviours depends on reputation management motives. When people are motivated to attain a good reputation, their self‐reported moral character may predict more hypocrisy by displaying stronger moral harshness towards others than towards themselves. Thus, claiming oneself as a moral person does not always translate into doing good deeds, but can manifest as showcasing one's morality to others. Desires for a positive reputation might help illuminate why self‐reported moral character often fails to capture real‐life moral decisions, and why (some) people who appear to be moral are susceptible to accusations of hypocrisy—for applying higher moral standards to others than to themselves.

## AUTHOR CONTRIBUTIONS

**Mengchen Dong:** Conceptualization; data curation; formal analysis; funding acquisition; investigation; methodology; project administration; resources; validation; visualization; writing – original draft; writing – review and editing. **Tom R. Kupfer:** Validation; writing – review and editing. **Shuai Yuan:** Formal analysis; methodology; validation; writing – review and editing. **Jan‐Willem Van Prooijen:** Funding acquisition; methodology; resources; supervision; validation; writing – review and editing.

## CONFLICT OF INTEREST

None.

### OPEN RESEARCH BADGES

This article has earned Open Data, Open Materials and Preregistered Research Design badges. Data, materials and the preregistered design and analysis plan are available at [https://osf.io/3f8br/?view_only=6bd6c46b26cb45588d43d755dab4b928; https://osf.io/75wrm/?view_only=48e6d0fe93274c319faa8d72705f6bf5].

## Supporting information


Appendix S1

## Data Availability

The data that support the findings of this study are openly available in Open Science Framework at https://doi.org/10.17605/OSF.IO/3f8br, Dong et al. (2021).

## Footnotes

^1^
Relatedly, though our earlier theorizing focused on moral character in general, we later found it more appropriate to discuss in the scope of *self‐reported* moral character since we examined moral character only with self‐reported measures.
