# Supplementary material for: Being good to look good: Self‐reported moral character predicts moral double standards among reputation‐seeking individuals
Source: Br J Psychol. 2022 Nov 4;114(1):244–61. doi: 10.1111/bjop.12608 (PMC10098708; doi:10.1111/bjop.12608)
Supplement: Supplementary file 1 — Appendix S1 [file BJOP-114-244-s001.docx]

Supplementary Materials for

**Being Good to Look Good:**

**Self-Reported Moral Character Predicts Moral Double Standards Among Reputation-Seeking Individuals**

Table of Contents

[Alternative Analyses for Study 2 2](#_Toc108352207)

[Pilot Study of Study 3 5](#_Toc108352208)

[Supplementary Figures 10](#_Toc108352209)

[Supplementary Tables 12](#_Toc108352210)

[Study 1 Materials 18](#_Toc108352211)

[Study 2 Materials 20](#_Toc108352212)

[Study 3 Materials 29](#_Toc108352213)

[Pilot Study of Study 3 Materials 35](#_Toc108352214)

# Alternative Analyses for Study 2

We performed factor analyses for both justice sensitivity and self-monitoring measures (see Supplementary Table 4 for a summary). First, confirmatory factor analyses suggested that the two-factor model was significantly better than the one-factor model (*Δχ^2^* = 117.81, *p* < .001). We therefore performed the alternative analyses with the mean scores of respectively perpetrator or observer sensitivity items as independent indicators of moral character. Second, we performed confirmatory factor analyses for both the one-factor and the three-factor model of the self-monitoring scale. We found a better fit of the three- rather than one-factor model (*Δχ^2^* = 396.57, *p* < .001). We therefore used the mean score of the 10 items within the other-directness dimension of self-monitoring as the indicator of reputation management motives in the alternative analyses.

We performed the alternative two-level linear mixed model, regressing moral blame (*M* = 4.54, *SD* = 1.89) on target (self = -1, other = 1), mean-centered moral character (perpetrator sensitivity: *M* = 3.09, *SD* = 1.07; observer sensitivity: *M* = 3.22, *SD* = 0.91), 10-item reputation management motives (*M* = 0.47, *SD* = 0.23), and their two-way and three-way interactions, with random intercepts for participant id, transgressive scenario, and moral blame item (see Supplementary Table 5 in the SM for details). We did not include the interaction terms between perpetrator and observer sensitivity since these two facets of justice sensitivity were highly correlated (*r* = 0.60) and their interactions may cause multicollinearity issues. We found a significant interaction between moral character in a perpetrator perspective and reputation management motives (*β* = -0.12, *p* = .037, 95% CI [-0.23, -0.01]). Moral character in a perpetrator perspective positively correlated with overall moral blame, only for people with weaker (-1 *SD*; *B* = 0.29, *SE* = 0.11, *t* = 2.76, *p* = .006, $\eta_{p}^{2}$ = 0.04) but not stronger (+1 *SD*; *B* = 0.11, *SE* = 0.09, *t* = 1.14, *p* = .254, $\eta_{p}^{2}$ = 0.01) reputation management motives. More importantly, the hypothesized three-way interaction was marginally significant for moral character in a perpetrator perspective (*β* = 0.14, *p* = .050, 95% CI [0.01, 0.28]) but not observer perspective (*β* = 0.02, *p* = .781, 95% CI [-0.13, 0.17]). None of the other correlations were significant (*p*s > .0.127).

Similar to our findings in the manuscript, moral character in a perpetrator perspective interacted with moral target for people who had high (+1 *SD*; *B* = 0.32, *SE* = 0.15, *t* = 2.15, *p* = .033, $\eta_{p}^{2}$ = 0.02) but not low (-1 *SD*; *B* = -0.25, *SE* = 0.15, *t* = -1.65, *p* = .100, $\eta_{p}^{2}$ = 0.01) reputation management motives. When reputation management motives were low (-1 *SD*), moral character predicted a stronger blame for the self (*B* = 0.40, *SE* = 0.17, *t* = 2.33, *p* = .021, $\eta_{p}^{2}$ = 0.03) but not for others (*B* = 0.18, *SE* = 0.12, *t* = 1.49, *p* = .138, $\eta_{p}^{2}$ = 0.01). When reputation management motives were high (+1 *SD*), instead, moral character was positively associated with moral blame for others (*B* = 0.26, *SE* = 0.12, *t* = 2.17, *p* = .031, $\eta_{p}^{2}$ = 0.02) but not the self (*B* = -0.04, *SE* = 0.14, *t* = 0.30, *p* = .767, $\eta_{p}^{2}$ < 0.001). Together, the alternative analyses conceptually replicated our main findings by showing how the associations between self-reported moral character and moral double standards were moderated by reputation management motives.

# Pilot Study of Study 3

**Method**

***Participants***

We predetermined to recruit 200 participants. A priori power analysis indicated a sample of *N* = 126, with 80% power to detect a medium-size (i.e., $\eta_{p}^{2}$ = .06 as in Study 2) effect of our intended three-way interaction in a linear regression at an alpha level of 0.05. Two hundred and one American participants (117 males and 84 females; *M*_age_ = 37.9 years, *SD* = 10.5) completed our experiment through the crowdsourcing platform TurkPrime.com (Peer et al., 2017), and were all included in further analyses.

***Design and Procedure***

Participants first indicated their moral character, operationalized as moral identity. The moral identity measure was the 5-item internalization subscale of the Self-Importance of Moral Identity Questionnaire (Aquino & Reed, 2002). Participants were asked to read nine adjectives (e.g., generous, fair) and rated self-importance of these characteristics (e.g., “It would make me feel good to have these characteristics”; on a 7-point scale ranging from 1 = *Strongly disagree* to 7 = *Strongly agree*; α = 0.73).

Participants then completed a short survey ostensibly about their work status^[[1]](#footnote-1)^. To measure reputation management motives, we adopted a validated measure of concern about social esteem and status in interaction tasks (Blader & Chen, 2011, 2012; e.g., “I find it important that others acknowledge my status” “I wish to have high status”; α = 0.91 across 10 items).

Participants were eventually enrolled in an online interaction game and were randomly assigned to one of two roles, described as either an Observer (*n* = 96) or a Distributor (*n* = 105). The online interaction game was an incentivized dictator game with third-party punishment (see the SM for detailed instructions). We first explained that Distributors should assign a $10 bonus between the self and another online Recipient, with two options: (1) $8 to the self and $2 to the Recipient or (2) $5 to the self and $5 to the Recipient^[[2]](#footnote-2)^. We then explained that Observers had a $5 bonus and could choose to deduct the Distributors’ payoff at a 1:3 ratio (that is, if the Observer pays $0.1, the Distributor loses $0.3 without influencing the Recipient’s outcome). Participants were then randomly assigned to their role of a Distributor or an Observer. After making a Distributor choice, Distributors indicated “How acceptable do you think your decisions were in the game?” (on a 7-point scale ranging from to -3 = *Completely unacceptable* to 3 = *Completely acceptable*). In the Observer role, participants reviewed all potential Distributor choices. For each potential choice, they indicated “How acceptable do you think the Distributor's decisions were in the game?” on the same scale, and indicated the amount they wanted to pay to reduce the Distributor’s bonus (from $0 to $2.5 with unit increments of $0.1). The Distributors and Observers were later matched into pairs, and 5% of them received the actual bonus as determined by their choices.

**Results**

Among the included Distributors (*N* = 102), 54 (52.9%) participants made an $8/$2 offer and 48 (47.1%) participants made a $5/$5 offer. We first analyzed Distributors’ actual behavior, and then contrasted their judgment of own behavior with Observers’ judgment of identical behavior.

***Moral Behavior***

We conducted a binary logistic regression to examine the effects of mean-centered moral character (*M* = 5.59, *SD* = 1.11) and reputation management motives (*M* = 3.80, *SD* = 1.63), and their interaction, on Distributors’ choice between $8/$2 (= 0) and $5/$5 (= 1). None of the effects of moral character (*z* = 1.46, *p* = .145), reputation management motives (*z* = -1.81, *p* = .070), or their interaction (*z* = 0.40, *p* = .693) were significant.

***Moral Judgment***

We contrasted judgments of a $8/$2 offer in a linear regression, as a function of moral character, reputation management motives, and moral target (i.e., Observers judging others whilst Distributors evaluating the self). We found significant main effects of moral character (*B* = -0.37, *SE* = 0.17, *t* = -2.16, *p* = .032, $\eta_{p}^{2}$ = .03, 95% CI [-0.71, -0.03]) and moral target (*B* = -1.08, *SE* = 0.18, *t* = -5.85, *p* < .001, $\eta_{p}^{2}$ = .19, 95% CI [-1.45, -0.72]). More importantly, a three-way interaction effect emerged (*B* = -0.31, *SE* = 0.13, *t* = -2.40, *p* = .018, $\eta_{p}^{2}$ = .04, 95% CI [-0.57, -0.05]). The interaction between moral character and target was significant when people were low (*B* = -0.84, *SE* = 0.39, *t* = -2.14, *p* = .034) but not high (*B* = -0.47, *SE* = 0.26, *t* = -1.77, *p* = .079) on reputation management motive. As shown in Supplementary Figure 2, when people were less motivated by reputation (-1 *SD*), their moral character predicted lower acceptability judgments of their own (i.e., Distributor; *B* = -0.95, *SE* = 0.47, *t* = -2.04, *p* = .043) but not others’ selfish choices (i.e., Observer; *B* = 0.12, *SE* = 0.30, *t* = 0.42, *p* = .677). In contrast, with high reputation management motives (+1 *SD*), people reporting a stronger moral character judged others’ (*B* = -0.64, *SE* = 0.25, *t* = -2.53, *p* = .012), but not their own (*B* < 0.01, *SE* = 0.33, *t* = 0.001, *p* = .999), selfish choice as less acceptable. Put differently, people with strong (vs. weak) reputation management motives judged others’ transgressions as more acceptable when they were low (-1 *SD*; other: *B* = 0.59, *SE* = 0.20, *t* = 2.98, *p* = .003; self: *B* = -0.01, *SE* = 0.33, *t* = -0.02, *p* = .986) but not high on moral character (+1 *SD*; other: *B* = 0.17, *SE* = 0.27, *t* = 0.64, *p* = .523; self: *B* = 0.52, *SE* = 0.41, *t* = 1.27, *p* = .205). None of the other correlations were significant (*p*s > .115). The moral target by moral character by reputation management motives three-way interaction effect only emerged in judgments of a selfish $8/$2 but not a non-selfish $5/$5 offer (*B* = -0.09, *SE* = 0.06, *t* = -1.54, *p* = .125, $\eta_{p}^{2}$ = .02, 95% CI [-0.21, 0.03]).

# Supplementary Figures

**Supplementary Figure 1**

*The highly skewed distribution of count data on own transgressive frequency (skewness = 2.58; left) and its log-transformation (skewness = 1.69; right)*

**
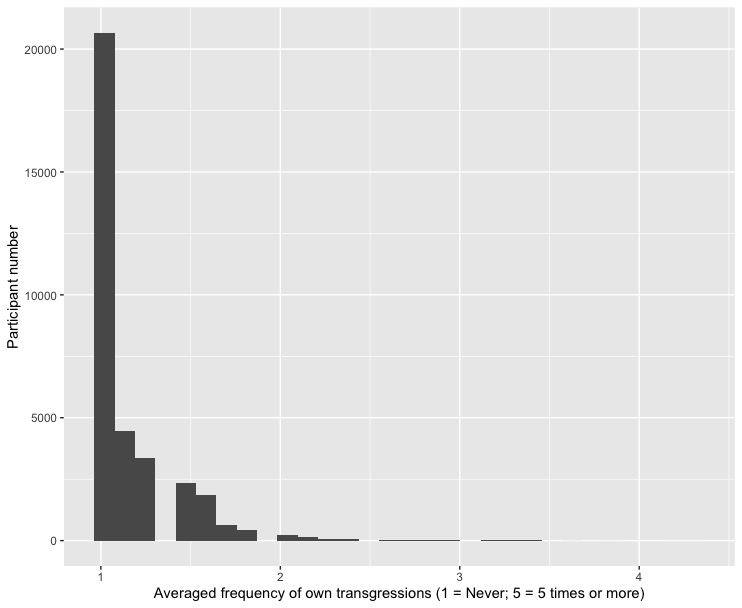

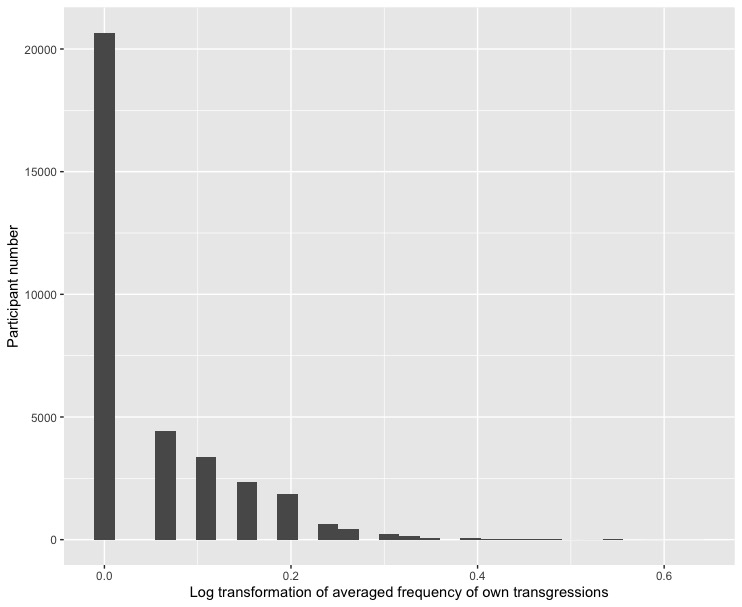
**

**Supplementary Figure 2**

*Moral acceptability judgments of own (= -1) and others’ (= 1) selfishness in an actual dictator game, as a function of moral character (as moral identity) and reputation management motives* (*as concern about status) in the Pilot Study of Study 3*


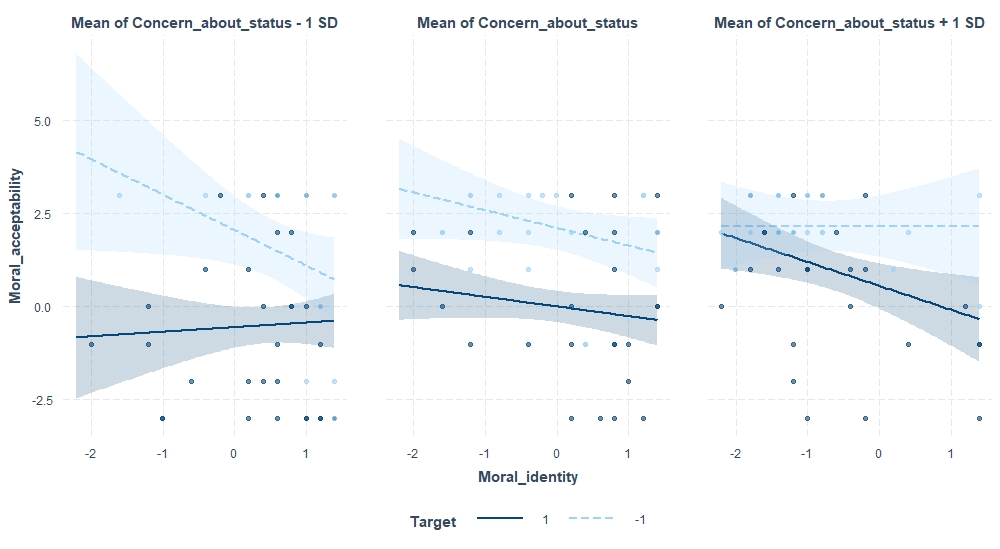


# Supplementary Tables

**Supplementary Table 1**

*The mixed effect models of (1) perceived wrongness of transgressions and (2) own frequency of transgressions, as a function of* *Benevolence and Universalism values (representing moral character), Power and Achievement values (representing reputation management motives), and the two-way interaction effect in Study 1*

|  | Wrongness of others’ transgressions | | | Frequency of own transgressions | | |
| --- | --- | --- | --- | --- | --- | --- |
| Random effects | Variance | *SD* |  | Variance | *SD* |  |
| id: country | 0.172 | 0.415 |  | < .001 | < .001 |  |
| Scenario | 0.191 | 0.437 |  | 0.025 | 0.158 |  |
| Residual | 0.320 | 0.566 |  | _ | _ |  |
| Fixed effects | *B* | *SE* | *t* | *B* | *SE* | *z* |
| Intercept | 3.268 | 0.219 | 14.95*** | 0.130 | 0.059 | 2.21* |
| Benevolence and Universalism (B+U) | 0.087 | 0.003 | 31.29*** | -0.031 | 0.002 | -15.91*** |
| Power and Achievement values (P+A) | -0.029 | 0.003 | -10.52*** | 0.026 | 0.002 | 13.54*** |
| (B+U) * (P+A) | 0.018 | 0.003 | 6.88*** | -0.01 | 0.002 | -7.43*** |

*Note.* **p* < .05. ****p* < .001.

**Supplementary Table 2**

*The alternative mixed effect models of own frequency of transgressions, as a function of Benevolence and Universalism values (representing moral character), Power and Achievement values (representing reputation management motives), and the two-way interaction effect in Study 1*

|  | Binomial GLMM  (non-transgression = 0;  any transgression = 1) | | | Poisson GLMM  (log-transformed own transgressive frequency) | | |
| --- | --- | --- | --- | --- | --- | --- |
| Random effects | Variance | *SD* |  | Variance | *SD* |  |
| id: country | 1.409 | 1.187 |  | 0.469 | 0.685 |  |
| Scenario | 1.750 | 1.323 |  | 2.235 | 1.495 |  |
| Fixed effects | *B* | *SE* | *z* | *B* | *SE* | *z* |
| Intercept | -3.706 | 0.723 | -5.13*** | -5.371 | 0.481 | -11.16*** |
| Benevolence + Universalism (B+U) | -0.309 | 0.011 | -28.68*** | -0.214 | 0.013 | -16.22*** |
| Power + Achievement values (P+A) | 0.232 | 0.011 | 21.06*** | 0.244 | 0.014 | 17.32*** |
| (B+U) * (P+A) | -0.122 | 0.010 | -12.07*** | -0.060 | 0.012 | -4.85*** |

*Note.* ****p* < .001.

**Supplementary Table 3**

*The mixed effect model of moral blame, as a function of one-factor justice sensitivity (representing moral character), 25-item self-monitoring (representing reputation management motives), moral target (self = -1 and other = 1), and the two-way and three-way interactions in Study 2*

|  | **Blame** | | | |
| --- | --- | --- | --- | --- |
| *Predictors* | *Estimates* | *CI* | *p* | *df* |
| (Intercept) | 4.45 | 3.50 – 5.41 | **<0.001** | 3.64 |
| Target | 0.17 | -0.05 – 0.38 | 0.137 | 176.00 |
| Justice sensitivity | 0.15 | -0.01 – 0.30 | 0.059 | 176.00 |
| Self-monitoring | 0.02 | -0.14 – 0.17 | 0.828 | 176.00 |
| Target * Justice sensitivity | 0.03 | -0.19 – 0.25 | 0.811 | 176.00 |
| Target * Self-monitoring | -0.04 | -0.26 – 0.17 | 0.682 | 176.00 |
| Justice sensitivity * Self-monitoring | -0.38 | -0.54 – -0.22 | **<0.001** | 176.00 |
| Target * Justice sensitivity * Self-monitoring | 0.51 | 0.27 – 0.75 | **<0.001** | 176.00 |
| **Random Effects** | | | | |
| σ^2^ | 2.90 | | | |
| τ_00_ _id_ | 0.33 | | | |
| τ_00_ _item_ | 0.02 | | | |
| τ_00_ _scenario_ | 0.39 | | | |
| Marginal R^2^ / Conditional R^2^ | 0.024 / 0.222 | | | |

**Supplementary Table 4**

*The confirmatory factor analyses* *of the measures of justice sensitivity (representing moral character) and self-monitoring (representing reputation motives) in Study 2*

| **Construct** | **Model** | ***df*** | *χ^2^* | ***RMSEA*** | ***CFI*** | ***TLI*** | *Δχ^2^* | ***p*** |
| --- | --- | --- | --- | --- | --- | --- | --- | --- |
| Justice sensitivity | One-factor | 170 | 607.83 | 0.12 | 0.71 | 0.68 |  |  |
|  | Two-factor | 169 | 490.02 | 0.10 | 0.79 | 0.76 | 117.81 | < .001 |
| Self-monitoring | One-factor | 275 | 610.75 | 0.08 | 0.59 | 0.55 |  |  |
|  | Three-factor | 166 | 214.18 | 0.04 | 0.94 | 0.93 | 396.57 | < .001 |

**Supplementary Table 5**

*The alternative mixed effect model of moral blame, as a function of Observer or Perpetrator justice sensitivity (representing moral character), 10-item self-monitoring (representing reputation management motives), moral target (self = -1 and other = 1), and the two-way and three-way interactions* *in Study 2*

|  | **Blame** | | | | | |  |  |
| --- | --- | --- | --- | --- | --- | --- | --- | --- |
| *Predictors* | *Estimates* | *CI* | | *p* | *df* | | |  |
| (Intercept) | 4.50 | | 3.55 – 5.45 | | **<0.001** | 3.67 | | |
| Target | 0.08 | | -0.14 – 0.31 | | 0.459 | 172.00 | | |
| Observer sensitivity | -0.03 | | -0.26 – 0.20 | | 0.805 | 172.00 | | |
| Perpetrator sensitivity | 0.18 | | -0.05 – 0.41 | | 0.127 | 172.00 | | |
| Self-monitoring | 0.04 | | -0.14 – 0.21 | | 0.680 | 172.00 | | |
| Target * Observer sensitivity | -0.02 | | -0.32 – 0.27 | | 0.881 | 172.00 | | |
| Target * Perpetrator sensitivity | 0.04 | | -0.24 – 0.33 | | 0.765 | 172.00 | | |
| Target * Self-monitoring | -0.13 | | -0.37 – 0.11 | | 0.290 | 172.00 | | |
| Observer sensitivity * Self-monitoring | -0.02 | | -0.27 – 0.22 | | 0.854 | 172.00 | | |
| Perpetrator sensitivity * Self-monitoring | -0.23 | | -0.44 – -0.01 | | **0.037** | 172.00 | | |
| Target * Observer sensitivity * Self-monitoring | 0.04 | | -0.25 – 0.33 | | 0.781 | 172.00 | | |
| Target * Perpetrator sensitivity * Self-monitoring | 0.27 | | 0.00 – 0.54 | | **0.050** | 172.00 | | |
| **Random Effects** | | | | | | |  |  |
| σ^2^ | 2.90 | | | | | |  |  |
| τ_00_ _id_ | 0.37 | | | | | |  |  |
| τ_00_ _item_ | 0.02 | | | | | |  |  |
| τ_00_ _scenario_ | 0.39 | | | | | |  |  |
| Marginal R^2^ / Conditional R^2^ | 0.017 / 0.225 | | | | | |  |  |

**Supplementary Table 6**

*The correlations between moral character and reputation management motives in Studies 1 to 3 and the Pilot Study in the SM*

|  | Study 1 | | Study 2 | | Study 3 | | Pilot Study | |
| --- | --- | --- | --- | --- | --- | --- | --- | --- |
|  | Power + Achievement | 10-item self-monitoring | | 25-item self-monitoring | | Status concern | | Status concern |
| Benevolence + Universalism | **0.162***** | **_** | | **_** | | **_** | | **_** |
| Perpetrator sensitivity | **_** | **-0.075** | | **-0.165** | | **_** | | **_** |
| Observer sensitivity | **_** | **-0.102** | | **-0.163** | | **_** | | **_** |
| Justice sensitivity (aggregated) | **_** | **-0.090** | | **-0.184** | | **_** | | **_** |
| Moral identity | **_** | **_** | | **_** | | **0.247***** | | **-0.509***** |

# Study 1 Materials



***The data of Study 1 was directly retrieved from the public datasets on the European Social Survey website. For more information about the measures, please check** [**https://www.europeansocialsurvey.org.***](https://www.europeansocialsurvey.org.*)

**[Self-as-target condition]**

• kptchng - Kept change from shop assistant/waiter when given too much, last 5 years
• payavtx - Paid cash with no receipt to avoid VAT or tax, last 5 years
• slcnsfl - Sold something second-hand and concealed its faults, last 5 years
• musdocm - Misused/altered card/document to pretend eligible, last 5 years
• flinsr - Made an exaggeration or false insurance claim, last 5 years
• pbofvr - Offered favour/bribe to public official for service, last 5 years
• flgvbnf - Falsely claim government benefit: social security or other, last 5 years



**[Other-as-target condition]**

• pyavtxw - Someone paying cash without receipt to avoid VAT or tax, how wrong
• slcnflw - Someone selling something second-hand and conceal faults, how wrong
• flinsrw - Someone making exaggerated/false insurance claim, how wrong
• pbofvrw - Public official asking favour/bribe in return for service, how wrong

**[Value questions; the used items are underlined]**

1. ipcrtiv - Important to think new ideas and being creative
2. imprich - Important to be rich, have money and expensive things
3. ipeqopt - Important that people are treated equally and have equal opportunities
4. ipshabt - Important to show abilities and be admired
5. impsafe - Important to live in secure and safe surroundings
6. impdiff - Important to try new and different things in life
7. ipfrule - Important to do what is told and follow rules
8. ipudrst - Important to understand different people
9. ipmodst - Important to be humble and modest, not draw attention
10. ipgdtim - Important to have a good time
11. impfree - Important to make own decisions and be free
12. iphlppl - Important to help people and care for others well-being
13. ipsuces - Important to be successful and that people recognize achievements
14. ipstrgv - Important that government is strong and ensures safety
15. ipadvnt - Important to seek adventures and have an exciting life
16. ipbhprp - Important to behave properly
17. iprspot - Important to get respect from others
18. iplylfr - Important to be loyal to friends and devote to people close
19. impenv - Important to care for nature and environment
20. imptrad - Important to follow traditions and customs
21. impfun - Important to seek fun and things that give pleasure

Coding scheme:

Benevolence 12, 18

Universalism 3, 8, 19

Achievement 4, 13

Power 2, 17

Hedonism 10, 21

# Study 2 Materials

**[Moral character: Schmitt et al., 2005]**

(Observer sensitivity)

It bothers me when someone gets something they don’t deserve.

I am upset when someone does not get a reward he/she has earned.

I cannot easily bear it when someone unilaterally profits from others.

I can’t forget it for a long time when someone else has to fix others’ carelessness.

It disturbs me when someone receives fewer opportunities to develop his/her skills than others.

I am upset when someone is undeservingly worse off than others.

It worries me when someone has to work hard for things that come easily to others.

I ruminate for a long time when someone is being treated nicer than others for no reason.

It gets me down to see someone criticized for things that are overlooked with others.

I am upset when someone is being treated worse than others.

(Perpetrator sensitivity)

It disturbs me when I receive what others ought to have.

I have a bad conscience when I receive a reward that someone else has earned.

I cannot easily bear to unilaterally profit from others. 3.38 1.40 .21 .09 .74 .60 .67 4. It worries me for a long time when others have to fix my carelessness. 3.55 1.38 .24 .14 .65 .50 .63 5. It makes me sad when I receive more opportunities than others to develop my skills. 2.43 1.52 .47 .01 .57 .54 .73 6. I feel guilty when I am better off than others for no reason. 2.34 1.53 .51 .00 .52 .53 .73 7. It bothers me when things come easily to me that others have to work hard for. 1.79 1.51 .59 –.03 .33 .46 .62 8. I ruminate for a long time about being treated nicer than others for no reason. 2.25 1.49 .57 .02 .48 .55 .73 9. It bothers me when someone tolerates things with me that other people are being criticized for. 2.52 1.46 .54 .01 .58 .63 .78 10. I feel guilty when I receive better treatment than others.

**[Reputation management motive: Snyder, 1974]**

I find it hard to imitate the behavior of other people. (R)

My behavior is usually an expression of my true inner feelings, attitudes, and beliefs. (R)

At parties and social gatherings, I do not attempt to do or say things that others will like. (R)

I can only argue for ideas which I already believe. (R)

I can make impromptu speeches even on topics about which I have almost no information.

I guess I put on a show to impress or entertain people.

When I am uncertain how to act in a social situation, I look to the behavior of others for cues.

I would probably make a good actor.

I rarely seek the advice of my friends to choose movies, books, or music. (R)

I sometimes appear to others to be experiencing deeper emotions than I actually am.

I laugh more when I watch a comedy with others than when alone.

In groups of people, I am rarely the center of attention. (R)

In different situations and with different people, I often act like very different persons.

I am not particularly good at making other people like me. (R)

Even if I am not enjoying myself, I often pretend to be having a good time.

I'm not always the person I appear to be.

I would not change my opinions (or the way I do things) in order to please someone else or win their favor. (R)

I have considered being an entertainer.

In order to get along and be liked, I tend to be what people expect me to be rather than anything else.

I have never been good at games like charades or improvisational acting. (R)

I have trouble changing my behavior to suit different people and different situations. (R)

At a party, I let others keep the jokes and stories going. (R)

I feel a bit awkward in company and do not show up quite as well as I should. (R)

I can look anyone in the eye and tell a lie with a straight face (if for a right end).

I may deceive people by being friendly when I really dislike them.

***The below materials were adapted from Weiss, Burgmer, and Mussweiler (2018)***

**[Self-as-target condition]**

**[Scenario 1]**

**Imagine you have promised your new co-worker to complete an important report within the next days. However, you have been asked to assist another team with organizing an event. You consider this new task important for your standing within the company. However, it means that you have less time than expected for the report.**

**In order to be able to assist the team, you consider asking a less qualified intern to complete the report without telling your new co-worker about who actually wrote the report.**

**To what extent do you think...**

I would probably have good reasons to delegate the report to the intern.

It would reflect poorly on me if I delegated the report to the intern.

The circumstances would justify that I delegate the report to the intern.

It would be a questionable choice on my part if I delegated the report to the intern.

**[Scenario 2]**

**Imagine that at a company event, you run into a friend that works in another department. Chatting with this friend about each other's work has often been fun and quite useful for you. Sometimes you share information with each other that has proven to be valuable for your careers. Your friend asks you what you are currently working on. You recently started a new, confidential project together with your new co-worker.**

**You would really like to tell your friend about this new project. Sharing this information could be useful for you in the long run. However, neither of you is supposed to talk about this project at the current stage.

To what extent do you think...**

I would probably have good reasons to share information about this confidential project with my friend.

It would reflect poorly on me if I shared information about this confidential project with my friend.

The circumstances would justify that I share information about the confidential project with my friend.

It would be a questionable choice on my part if I shared information about the confidential project with my friend.

**[Scenario 3]**

**Imagine you had an interview for an exciting job at another company. The interview is scheduled during working hours and you do not want to postpone it, because you are quite enthusiastic about the possibility of working at the other firm.**

**Unfortunately, you and your new co-worker will both be very busy completing an important project during the next weeks as the deadline is approaching.**

**For these reasons, neither of you can take time off officially at this point; To be able to seize the promising chance at the other firm nevertheless, you think about calling in sick for the day of the interview.**

**To what extent do you think...**

I would probably have good reasons to call in sick to make it to the interview.

It would reflect poorly on me if I called in sick to make it to the interview.

The circumstances would justify that I call in sick to make it to the interview.

It would be a questionable choice on my part if I called in sick to make it to the interview.

**[Scenario 4]**

**Imagine that your company has a strict policy to implement regular backups in order to prevent data loss. These backups can be quite time consuming, and you are currently extremely busy completing your favorite side project.**

**Despite your obligation to do so, you consider postponing the backup of a task that you and your co-worker have been working on so that you can devote more time to completing the side project first.**

**To what extent do you think...**

I would probably have good reasons to postpone the backup.

It would reflect poorly on me if I postponed the backup.

The circumstances would justify that I postponed the backup.

It would be a questionable choice on my part if I postponed the backup.

**[Other-as-target condition]**

**[Scenario 1]**

**Imagine your new co-worker has promised you to complete an important report within the next days. However, he/she has been asked to assist another team with organizing an event. Your new co-worker considers this new task important for his/her standing within the company. However, it means that he/she has less time than expected for the report.**

**In order to be able to assist the team, your new co-worker considers asking a less qualified intern to complete the report without telling you about who actually wrote the report.**

**To what extent do you think...**

My new co-worker would probably have good reasons to delegate the report to the intern.

It would reflect poorly on my new co-worker if he/she delegated the report to the intern.

The circumstances would justify that my new coworker delegates the report to the intern.

It would be a questionable choice on my new coworker's part if he/she delegated the report to the intern.

**[Scenario 2]**

**Imagine that at a company event, your new co-worker runs into a friend that works in another department. Chatting with this friend about each other's work has often been fun and quite useful for your new co-worker. Sometimes they share information with each other that has proven to be valuable for their careers.**

**The friend asks your new co-worker what he/she is currently working on. He/she recently started a new, confidential project together with you.**

**Your new co-worker would really like to tell his/her friend about this new project. Sharing this information could be useful for him/her in the long run. However, neither of you is supposed to talk about this project at the current stage.**

**To what extent do you think...**

My new co-worker would probably have good reasons to share information about this confidential project with his/her friend.

It would reflect poorly on my new co-worker if he/she shared information about this confidential project with his/her friend.

The circumstances would justify that my new coworker shares information about the confidential project with his/her friend.

It would be a questionable choice on my new coworker's part if he/she shared information about the confidential project with his/her friend.

**[Scenario 3]**

**Imagine your new co-worker had an interview for an exciting job at another company. The interview is scheduled during working hours and he/she does not want to postpone it, because he/she is quite enthusiastic about the possibility of working at the other firm.**

**Unfortunately, your new co-worker and you will both be very busy completing an important project during the next weeks as the deadline is approaching.**

**For these reasons, neither of you can take time off officially at this point; To be able to seize the promising chance at the other firm nevertheless, your new co-worker thinks about calling in sick for the day of the interview.**

**To what extent do you think...**

My new co-worker would probably have good reasons to call in sick to make it to the interview.

It would reflect poorly on my new co-worker if he/she called in sick to make it to the interview.

The circumstances would justify that my new coworker calls in sick to make it to the interview.

It would be a questionable choice on my new coworker's part if he/she called in sick to make it to the interview.

**[Scenario 4]**

**Imagine that your company has a strict policy to implement regular backups in order to prevent data loss. These backups can be quite time consuming, and your new co-worker is currently extremely busy completing his/her favorite side project.**

**Despite his/her obligation to do so, your new co-worker considers postponing the backup of a task that he/she and you have been working on so that he/she can devote more time to completing the side project first.**

**To what extent do you think...**

My new co-worker would probably have good reasons to postpone the backup.

It would reflect poorly on my new co-worker if he/she shared information about this confidential project with his/her friend.

The circumstances would justify that my new coworker shares information about the confidential project with his/her friend.

It would be a questionable choice on my new coworker's part if he/she shared information about the confidential project with his/her friend.

# Study 3 Materials

**[Moral character: Aquino & Reed, 2002]**

Listed below are some characteristics that might describe a person:

Caring, Compassionate, Fair, Friendly, Generous, Helpful, Hardworking, Honest, and Kind

The person with these characteristics could be you or it could be someone else. For a moment, visualize in your mind the kind of person who has these characteristics. Imagine how that person would think, feel, and act. When you have a clear image of what this person would be like, answer the following questions.

It would make me feel good to be a person who has these characteristics.

Being someone who has these characteristics is an important part of who I am.

I would be ashamed to be a person who had these characteristics. (R)

Having these characteristics is not really important to me. (R)

I strongly desire to have these characteristics.

**[Reputation management motive: Blader & Chen, 2011]**

I am rarely concerned how my status compares to others. (R)

I do not consider what others think about my status. (R)

I react very negatively when my status is challenged.

I am very sensitive to whether I feel my status is being threatened during my interactions with others.

I find it important that others acknowledge my status.

I try hard to maintain my status in my interactions with others.

I find it upsetting when others do not seem to think the same of my status as I do.

It is important to me that others agree with me about my status.

I wish to have high status.

When I feel my status is low, I feel very bad.

**[Economic game instructions]**

In addition to the basic pay you will receive for completing this study, you may also win extra money ($0 ~ $8) as a bonus, specifically as below:

You will participate in an interaction game with other Prolific workers. The game has three roles: **Distributor, Recipient, and Observer**.

All participants are organized into three-player groups, and 1 out of every 20 groups will receive the amount of money as indicated in the game. That is to say, each participant has a 5% chance to win the actual bonus, which may also depend on the decisions of other players in the same group.

On the next pages, you will (1) be introduced to ALL the roles, (2) be assigned to your role, and (3) make decisions based on your role.

**--Below is information about the Distributor role--**

The Distributor assigns $10 bonus between the self and another Recipient.

The Distributor has two choices:

(1) Keep $8 and give $2 to the Recipient

(2) Keep $5 and give $5 to the Recipient

**--Below is information about the Recipient role--**

After the Distributor makes a choice between (1) $8/$2 and (2) $5/$5, the Recipient receives either $2 or $5 correspondingly.

The Recipient cannot make changes to the Distributor’s choice or payoff.

**--Below is information about the Observer role--**

The Observer receives a fixed bonus of $3.

The Observer knows the choice of the same-group Distributor, but cannot make changes to the payoffs of the Distributor and the Recipient.

After reading the above instructions, please answer four questions accordingly:

Can Distributors choose between (1) $8/$2 and (2) $5/$5 at their own will?

Yes (1)

No (2)

Not sure (3)

Validation: (when choosing other than “Yes”) Your answer is not correct. Distributors can choose between (1) $8/$2 and (2) $5/$5 at their own will.

Can Recipients make changes to Distributions’ payoff?

Yes (1)

No (2)

Not sure (3)

Validation: (when choosing other than “No”) Your answer is not correct. Recipients cannot make changes to Distributions’ payoff.

Can Observers make changes to the payoffs of Distributors and Recipients?

Yes (1)

No (2)

Not sure (3)

Validation: (when choosing other than “No”) Your answer is not correct. Observers cannot change the payoffs of the Distributors and the Recipients.

Which statement best describes the Observers’ payoff?

Observers’ payoff depends on Distributor’s choice (1)

Observers’ payoff depends on how they interact with Distributors (2)

Observers receive a fixed bonus of $3 (3)

Validation: (when choosing other than “Observers receive a fixed bonus of $3”) Your answer is not correct. Observers receive a fixed bonus of $3.

**[Participants playing the Distributor role]**

**--You are assigned to be a Distributor --**

What is your role in this interaction game?

Distributor (1)

Recipient (2)

Observer (3)

Validation: (when choosing other than Distributor) Your answer is not correct. You are a Distributor in this game.

**-- Now click on “→” to make your Distributor choice --**

How do you want to distribute $10?

Keep $8 and give $2 to the Recipient

Keep $5 and give $5 to the Recipient

*NOTE: The other players are REAL. Your choice will have a chance to determine how much bonus you and another Recipient actually receive. Your decision from this page will be used to calculate your bonus and the bonus of the Recipient.*

[page break]

How acceptable do you think your choice is in the game?

(-3 = *Completely unacceptable* to 3 = *Completely acceptable*)

**[Participants playing the Observer role]**

**--You are an Observer in this game—**

What is your role in this interaction game?

Distributor (1)

Recipient (2)

Observer (3)

Validation: (when choosing other than Observer) Your answer is not correct. You are an Observer in this game.

**-- Now click on “→” to make your Observer choices –**

Please note that we do not yet know the actual Distributor choice in your group. However, there are two possible choices in total.

As an Observer, you will see and evaluate both possibilities on the next two pages. One of the two possibilities will be the Distributor's actual choice in your group.

If the Distributor in your group chooses to keep $8 and give the Recipient $2, instead of keeping $5 and giving the Recipient $5, how acceptable do you think the Distributor's choice is in the game?

(-3 = *Completely unacceptable* to 3 = *Completely acceptable*)

If the Distributor in your group chooses to keep $5 and give the Recipient $5, instead of keeping $8 and giving the Recipient $2, how acceptable do you think the Distributor's choice is in the game?

(-3 = *Completely unacceptable* to 3 = *Completely acceptable*)

*NOTE: The other players are REAL. The Distributor’s choice will have a chance to determine how much bonus the Distributor and another Recipient actually receive.* *The Distributor’s actual decision will be used to calculate both the Distributor’s and the Recipient’s bonus.*

# Pilot Study of Study 3 Materials

In addition to the basic pay you will receive for completing this study, you may also win extra money ($0~$8) as a bonus, specifically as below:

You will participate in an interaction game with other Mturk workers. The game has three Players: **Distributor, Recipient, and Observer**.

All participants are organized into three-player groups, and each participant has a 5% chance to win the actual bonus, as also determined by players in the same group. In other words, one among every twenty groups will receive the amount of money as indicated in the game.

**--Below is information about Distributor role--**

Each distributor assigns $10 bonus between the self and another recipient.

Each Distributor has two options:

(1) Giving $8 to the self and $2 to a Recipient

(2) Giving $5 to the self and $5 to a Recipient

Distributor could choose from:

(1) $8/$2 and (2) $5/$5 at **his/her own will, OR**

(3) **guess two online six-sided dice ONCE** to help him/her decide, one dice in GREEN and the other in RED.

The dice guessing rules are:

1. IF a Distributor guesses BOTH the GREEN and the RED dice CORRECTLY (with a 1/36 chance), the Distributor should (1) give $8 to the self and $2 to the Recipient
2. IF a Distributor guesses ANY of the two dice INCORRECTLY (with a 35/36 chance), the Distributor should (2) give $5 to the self and $5 to the Recipient

IF a Distributor chooses to guess the dice to decide between (1) $8/$2 and (2) $5/$5, the Distributor FIRST guesses the numbers in his/her head, THEN roll the two dice once, and LASTLY indicate on one question whether he/she guesses correctly.

After reading the above instructions, please answer four questions accordingly:

Are Distributors free to choose by themselves, instead of guessing the dice, if they want?

Yes (1)

No (2)

Not sure (3)

Validation: (when choosing other than Yes) Your answer is not correct. Distributors are free to choose from (1) $8/$2, or (2) $5/$5, and (3) guessing the dice.

According to the dice guessing rules, if a Distributor guesses 6 for the GREEN dice and 3 for the RED dice, but the dice rolling shows 3 for the GREEN dice and 6 for the RED dice, should the Distributor get $8 for the self and $2 for the Recipient?

Yes (1)

No (2)

Not sure (3)

Validation: (when choosing other than No) Your answer is not correct. Distributors should gain $8 only when they guess BOTH the green and the red six-sided dice correctly.

What is the chance to win $8 for the self if a Distributor choose to guess the dice?

1/6 (≈ 16.7%) (1)

1/9 (≈ 11.1%) (2)

1/12 (≈ 8.3%) (3)

1/36 (≈ 2.8%) (4)

Validation: (when choosing other than 1/36) Your answer is not correct. Distributors should gain $8 only when they guess both the green and the red six-sided dice correctly (i.e., 1/36 chance).

Are Distributors required to write down the numbers that they guess before they roll the dice?

Yes (1)

No (2)

Not sure (3)

Validation: (when choosing other than No) Your answer is not correct. Distributors guess the numbers in their head before rolling the dice.

**--Below you try Distributor role once as exercise--**

How do you want to distribute $10?

$8 to the self and $2 to a recipient

$5 to the self and $5 to a recipient

Guess two dice

[If choosing “Guess two dice”]

What is the GREEN dice? And what is the RED dice?

[Javascript of dice rolling]

Did you guess both the dice correctly?

YES! ---Congrats! You get $8 for yourself and $2 for a recipient

NO! ---Not bad! You get $5 for yourself and $5 for a recipient

Note: This is an exercise session, to have ALL participants experience how Distributors make choices. Your answers will NOT be used to calculate your actual bonus.

**--Below is information about Recipient role--**

After a Distributor makes a choice between (1) $8/$2 and (2) $5/$5, a Recipient receives either $2 or $5 correspondingly.

**--Below is information about Observer role--**

Each Observer receives a fixed $5.

An Observer knows the choices of the same-group Distributor. An example is shown as below:


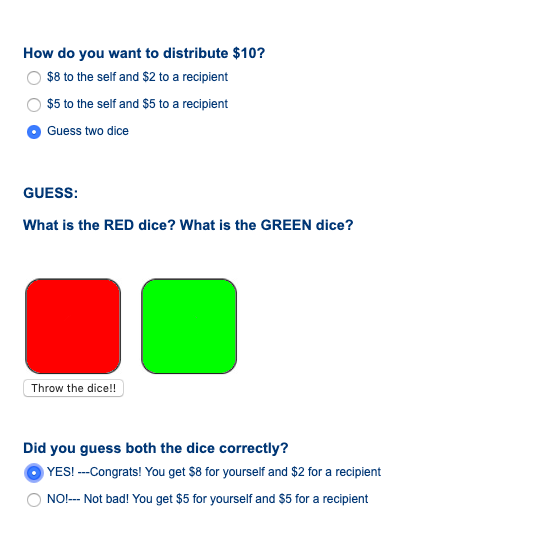


The Observer then decides whether to use his/her own $5 to reduce the Distributor’s bonus at a ratio of 1: 3.

That is to say, if an Observer decides to use X cents to reduce the Distributor’s bonus, X cents are subtracted from the Observer’s bonus, 3X cents are subtracted from the Distributor’s bonus. But the Observer’s decision does not change the Recipient’s bonus.

After reading the above instructions, please answer four questions accordingly:

If a Distributor gets $8/$2 without guessing the dice. A Observer then uses $1.3 to reduce the Distributor’s bonus. How much will the Distributor eventually get?

$8 (1)

$4.1 (2)

$3.1 (3)

$2 (4)

Validation: (when choosing other than $4.1) Your answer is not correct. The Distributor gets $\$8-(\$1.3\times3)=\$4.1$.

If a Distributor gets $5/$5 after indicating to have guessed the dice incorrectly. An Observer then uses $0.1 to reduce the Distributor’s bonus. How much will the Distributor eventually get?

$8 (1)

$5 (2)

$4.7 (3)

$0.3 (4)

Validation: (when choosing other than $4.1) Your answer is not correct. The Distributor gets $\$5-(\$0.1\times3)=\$4.7$.

**[Participants playing the Distributor role]**

**--You are a Distributor in this game—**

What is your role in this interaction game?

Distributor (1)

Recipient (2)

Observer (3)

Validation: (when choosing other than Distributor) Your answer is not correct. You are a Distributor in this game.

**-- Now click on “→” to make your Distributor choice –**

How do you want to distribute $10?

$8 to the self and $2 to a recipient

$5 to the self and $5 to a recipient

Guess two dice

[If choosing “Guess two dice”]

What is the GREEN dice? And what is the RED dice?

[Javascript of dice rolling]

Did you guess both the dice correctly?

YES! ---Congrats! You get $8 for yourself and $2 for a recipient

NO! ---Not bad! You get $5 for yourself and $5 for a recipient

How acceptable do you think your decisions were in the game?

(-3 = *Completely unacceptable* to 3 = *Completely acceptable*)

*NOTE: The other players are REAL, and your decision will have a chance to determine how much bonus you and the other players actually receive. Your decision from this page will be used to calculate your bonus and the bonus of the other players.*

**[Participants playing the Observer role]**

**--You are an Observer in this game—**

What is your role in this interaction game?

Distributor (1)

Recipient (2)

Observer (3)

Validation: (when choosing other than Observer) Your answer is not correct. You are an Observer in this game.

**-- Now click on “→” to make your Observer choices --**

If the Distributor in your group chooses as below...

[Screenshot of selfish behavior]


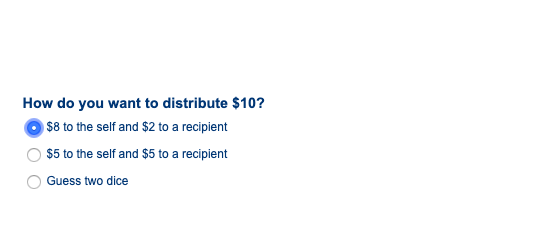


How acceptable do you think the Distributor's decisions were in the game?

(-3 = *Completely unacceptable* to 3 = *Completely acceptable*)

You have a $5 bonus. How much do you want to pay, to reduce the Distributor’s bonus at a 1:3 ratio (i.e., if you pay $0.1, the Distributor loses $0.3)? ($0 to $2.5)

*NOTE: The other players are REAL, and your decision will have a chance to determine how much bonus you and the other players actually receive. Your decision from this page will be used to calculate your bonus and the bonus of the other players.*

If the Distributor in your group chooses as below...

[Screenshot of fairness behavior]


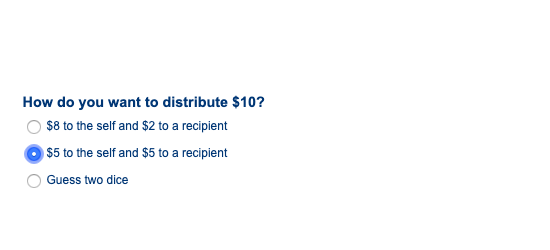


How acceptable do you think the Distributor's decisions were in the game?

(-3 = *Completely unacceptable* to 3 = *Completely acceptable*)

You have a $5 bonus. How much do you want to pay, to reduce the Distributor’s bonus at a 1:3 ratio (i.e., if you pay $0.1, the Distributor loses $0.3)? ($0 to $2.5)

*NOTE: The other players are REAL, and your decision will have a chance to determine how much bonus you and the other players actually receive. Your decision from this page will be used to calculate your bonus and the bonus of the other players.*

1. There was another 8-item scale measuring participants’ current status at work (Yu et al., 2019; e.g., “In general, my position tends to be highly respected”; α = 0.92), which was not significantly associated with moral acceptability judgment (*t* = 1.83, *p* = .07), and was thus not included in the main analyses. [↑](#footnote-ref-1)
2. An online dice rolling procedure was also introduced to help Distributors choose between (1) $8/$2 and (2) $5/$5. However, very few Distributors (*n* = 3) chose to roll the dice. We therefore excluded this category from further analyses and mainly focused on participants who chose $8/$2 or $5/$5 directly. [↑](#footnote-ref-2)
